# Distinct distribution and responses of IgM^+^, IgT1^+^ and IgT2^+^ B cells in common carp

**DOI:** 10.3389/fimmu.2024.1490776

**Published:** 2024-11-11

**Authors:** Awatif Eltijani, Carmen W. E. Embregts, Susana Magadan, Jingjing Wang, Sylvia Brugman, Pierre Boudinot, Geert F. Wiegertjes, Maria Forlenza

**Affiliations:** ^1^ Aquaculture and Fisheries Group, Department of Animal Sciences, Wageningen University & Research, Wageningen, Netherlands; ^2^ Cell Biology and Immunology Group, Department of Animal Sciences, Wageningen University & Research, Wageningen, Netherlands; ^3^ Immunology Laboratory, Research Centre for Nanomaterials and Biomedicine (CINBIO), Universidade de Vigo, Vigo, Spain; ^4^ School of Marine Science and Engineering, Qingdao Agricultural University, Qingdao, China; ^5^ Host-Microbe Interactomics Group, Department of Animal Sciences, Wageningen University & Research, Wageningen, Netherlands; ^6^ French National Institute for Agriculture, Food and Environment (INRAE), Department of Virology and Molecular Immunology, Jouy-en-Josas, France

**Keywords:** teleosts, IGT, IgM, mucosal B cells, systemic B cells

## Abstract

In teleosts, the immunoglobulin classes produced by B cells are IgM, IgD, and IgT/IgZ. IgT was initially described as an immunoglobulin specialized in mucosal responses; accumulating evidence, however, shows that it is also involved in systemic immune responses. Two types of IgT/IgZ (IgT1 and IgT2) were previously described in common carp, but their further characterization was hampered by the lack of specific tool. In the current study, we developed and validated polyclonal antibodies against carp IgT1 and IgT2 and used them in combination with well validated monoclonal antibody against carp IgM (WCI12), to study the distribution of IgM^+^, IgT1^+^ and IgT2^+^ B cells or their secreted immunoglobulins in various mucosal and systemic organs of carp. Finally, we also preliminary assessed the B cell response to infection with the blood-borne parasite *Trypanoplasma borreli.* Using these tools, we report on the distinct expression of soluble immunoglobulins in systemic and mucosal compartments. IgT1 and IgM were expressed in mucosal as well as systemic organs and responded to systemic parasitic infection, whereas IgT2 was preferentially expressed at mucosal sites and did not respond to systemic infections. By studying the distribution of B cells in different organs, compartmentalization of the three B cell subtypes was observed in gills and gut, whereas splenic B cells appeared as organized clusters around ellipsoids. Our results provide insights into the distribution and to some extent the function of B cells in carp, indicating that our newly developed tools are valuable for future studies aiming at the further characterization of immune responses of carp to infections and vaccination.

## Introduction

B cells are the main mediators of adaptive humoral immunity, they produce immunoglobulins that play a prominent role in both innate and adaptive immunity and are involved in protecting the organism from a wide variety of pathogens. The main classes of immunoglobulins that are produced by B cells in teleost fish are IgM (1), IgD (2), and the more recently identified IgT/IgZ (3, 4). The *igh* locus encoding these three immunoglobulins (μ, δ, and τ/ζ) is arranged in a translocon organization. In this organization, the VH gene segments are shared by the different *igh* genes and are followed by the locus encoding IgT (Dτ-Jτ-Cτ), and IgM and IgD (D_μ/δ_-J_μ/δ_-C_μ/δ_). Alternatively, the Dτ-Jτ-Cτ cluster of *IgT* can also be located within the set of VH gene segments. Within an haplotype, such organization results in the exclusive expression of either IgM/IgD or IgT at a given locus; this is because recombination between VH and the D_μ/δ_-J_μ/δ_-C_μ/δ_ deletes Dτ-Jτ-Cτ (3, 4). The IgT locus is unique to teleosts but highly diverse among them, and variable numbers of gene copies were reported in different fish species. As much as four functional genes have been reported in stickleback (5), three functional genes were reported for rainbow trout (6) and Atlantic salmon (7), two genes were found in zebrafish (8) and common carp (9), while only a single functional gene has been reported for fugu (10). More diversity among teleosts is added by a variable number of encoded immunoglobulin constant heavy domains between species. Generally, the constant region of the heavy chain of IgT is composed of four immunoglobulin domains (3, 11, 12), but three domains have been reported in stickleback (5), Antarctic fish (13) and spotted gar (14), while only two domains were reported in fugu (10) and IgT2 of common carp (15). While carp *IgHT2* encodes two immunoglobulin domains (CH1τ_2_-CH2-τ_2_), carp *IgHT1* encodes four domains (CH1-4τ_1_). The first constant domain of IgT2 (CH1τ_2_) is highly similar to CH1μ of carp IgM, and its second constant domain (CH2τ_2_) shows high similarity to CH4τ1 of IgT1. Due to these similarities, *IgHT2* was initially described as a chimera (15), but based on genetic studies in the aforementioned teleost species and the release of the common carp genome (16, 17) we now know that the IgT2 isotype is encoded by a separate gene in carp. Interestingly, unlike IgM and IgD, IgT is not present in all fish species. Recently, it was reported that IgT is absent in 23 out of 73 species that were investigated (14). This includes channel catfish (18) and medaka (19).

Owing to the availability of monoclonal antibodies against the different Ig heavy chains, four patterns of Ig expression by naïve B cells were reported, at least in rainbow trout. Three subsets are expressing a single immunoglobulin isotype on their surface, either IgM (IgM^+^IgD^-^ IgT^-^), IgD (IgM^-^IgD^+^IgT^-^) (20–23), or IgT (IgM^-^IgD^-^ IgT^+^) (24), while the fourth subset expresses IgM and IgD (IgM^+^IgD^+^IgT^-^) (23, 25). Functional studies have initially described IgT as an immunoglobulin dedicated to mucosal immune responses. Studies conducted in rainbow trout have described IgT as immunoglobulin dedicated to mucosal immunity in the intestine (25), skin (26, 27), nasal cavity (28), gills (29), pharyngeal mucosa (30) and buccal cavity (31). These studies have demonstrated the importance of IgT in immune response against parasitic (25, 26, 30) and bacterial (27) infections. Moreover, it was shown that IgT is the predominant immunoglobulin that coats commensal bacteria at these mucosal sites (25, 26, 30). The above-mentioned studies have also shown the presence of pathogen-specific IgT titers in serum, even though they were significantly lower than in mucus. However, accumulating evidence suggests IgT involvement in systemic immune responses as well (20). Studies conducted in zebrafish have shown high levels of IgZ1 in serum seven days after bacterial infection (32). Similarly, gene expression studies conducted in turbot indicated that intraperitoneal administration of formalin-inactivated bacteria, induced the expression of membrane IgT in systemic organs such as the spleen and head kidney (33). In addition, IgT^+^ B cells in trout, have been shown to respond to intramuscular DNA vaccination against viral hemorrhagic septicemia virus (VHSV) by infiltrating to the site of injection (34). Moreover, in trout infected with the parasite causing proliferative kidney disease, *IgT* was upregulated, IgT^+^ B cells proliferated in the head kidney, and parasites were coated by IgT (35). A recent study in zebrafish also reported on the maternal transfer of IgT likely playing a role in protection of the offspring during early life (36). All these studies suggest that IgT plays important functions beyond mucosal barriers. In common carp, transcriptional analysis has shown the upregulation of *IgHT1* after infection with the systemic extracellular blood parasite *Trypanoplasma borreli (T. borreli)* and upregulation of *IgHT2* after infection with the skin parasite *Lernaea* (9).

In the present study, we developed and validated polyclonal antibodies against carp IgT1 and IgT2. We used these antibodies in combination with a previously established and well validated monoclonal antibody against carp IgM (WCI12 (37),) to evaluate the presence of the respective secreted immunoglobulins in the serum, mucus and on mucus-associated bacteria. We also studied the relative distribution of IgT1^+^, IgT2^+^ and IgM^+^ B cells in the gills, gut, spleen and head kidney of healthy carp and preliminarily assessed the responses of the different B cell subsets to a systemic infection with the blood parasite *T. borreli* by analyzing the presence of parasite-specific antibodies in serum and B cell proliferation and distribution in spleen, one of the most affected organs by this infection. Altogether, our results provide insights into the tissue distribution of B cell subsets in carp and the involvement of IgT in responses to systemic infections (38).

## Materials and methods

### Animals

In Eurasia there are two (poorly defined) sub-species of common carp; *Cyprinus carpio carpio* and *Cyprinus carpio haematopterus*, the first is referred to as European, the second as East-Asian common carp (39). In this study, European common carp (*Cyprinus carpio carpio*) between 6 and 11 months of age, originated from a cross between the Hungarian R8 and the Polish R3 strain (40) were used. Carp were bred in the Aquatic Research Facility (ARF) of Wageningen University (Carus), raised at 20-23 °C in recirculating UV-treated water and fed pelleted dry feed (Skretting, Nutreco) twice daily. Experiments were performed with the approval of animal experiment committee (DEC) of Wageningen university under project number 2017.W-0034.

### Cloning of *IgT1* and *IgT2* from European common carp

The sequences corresponding to the published (East-Asian) common carp *IgHZ1* (Acc. Number AB598367) and *IgHZ2 (*Acc. Number AB598368) (9), were used for blast analyses of the European common carp genome and transcriptome (16, 17). On genomic scaffold 44259 we retrieved sequences with high similarity to the published *IgHZ2*, whereas on scaffold 44206, four exons encoding immunoglobulin constant (CH) domains were identified of which three (CH2, CH3, and CH4) had high similarity to the published *IgHZ1*; a fourth exon had strong relationship to the CH1 domains of other *IgHZ* sequences (from zebrafish and from the recently reported *IgHZ3* of East-Asian common carp) but had low sequence similarity to the previously reported *IgHZ1*. To confirm the *IgHZ1* and *IgHZ2* sequences of European common carp, from now on referred to as *IgHT1* and *IgHT2* to comply with the original and reproposed nomenclature (3, 41), we designed forward primers specific for representative subgroups of carp VH domains as well as forward and reverse primers internal to the predicted CH domains of either *IgHT1* or *IgHT2* (**Table 1**). Total RNA was isolated from RNAlater-stored spleen tissue using the RNeasy mini kit (QIAgen) with on column DNase digestion. Total RNA (250-500 ng/reaction) was used as template for SuperScript^®^ One-Step RT-PCR System with Platinum^®^ *Taq* DNA Polymerase (Invitrogen) in combination with 200 nM of various combinations of forward and reverse primers (**Table 1**) up to a final volume of 50 μl. The RT-PCR reaction ran as follows: 94°C for 2 min, followed by 35 cycles at 94°C for 30 sec, 54-58°C for 30 sec, and 72°C for up to 2.5 min and a final elongation step at 72°C for 7 min. RT-PCR products were analysed on agarose gel, purified and cloned in pGEM-T easy plasmid (Promega). After transformation of JM109 competent *Escherichia coli*, on ampicillin (100 µg/ml) agar plates, more than 16 colonies for each product were selected and sent for sequencing (BaseClear).

**Table 1:** Primers used in this study.

Primers	Primer sequence
VhFam1_Fw1	GTCAGTAGTTGTGTCTCCAGGTGC
VhFam2_Fw1	TGGCTCTGGATTTACATTCAG
VhFam3_Fw1	CAGTGGTCATTAAACCTGGAGG
VhFam4_Fw1	CCATCCCATCAAGTCCTGTCACC
VhFam5_Fw1	GTTGGATCAGTCACCTGCTGAG
VhFam6_Fw1	TCGGTGAGGTTGTCCTGTCA
VhFam7_Fw1	CCCAGACTGATTCTGTGGTCTTA
VhFam8_Fw1	GACTGTCCGTCCTGATGCCAC
IgHT1_CH2_RV1	CCACATCCCAGTTGTCACT
IgHT1_CH1_RV2	CCAATCGCTTTTACTAATG
IgHT1_CH1_FW1	GATTCTACTGGGTCCTTCA+T
IgHT1_CH4_RV	TGGTAACAGTGGGCTTATT
IgHT2_CH2_RV1	GGGTCTCTCATGTGTGTGAG
IgHT2_CH1CH2_RV2	GCAATTTTAACCTCTAGTGAG
IgHT2_CH1_FW1	TAAATGGACGGATTATG+CTAAG
IgHT2_CH2_RV2	TTAATCACATGATAATTCTGG

+ indicates locked nucleic acid modification (LNA).

### Prokaryotic expression and protein purification of IgT1 and IgT2

**Figure 1:**
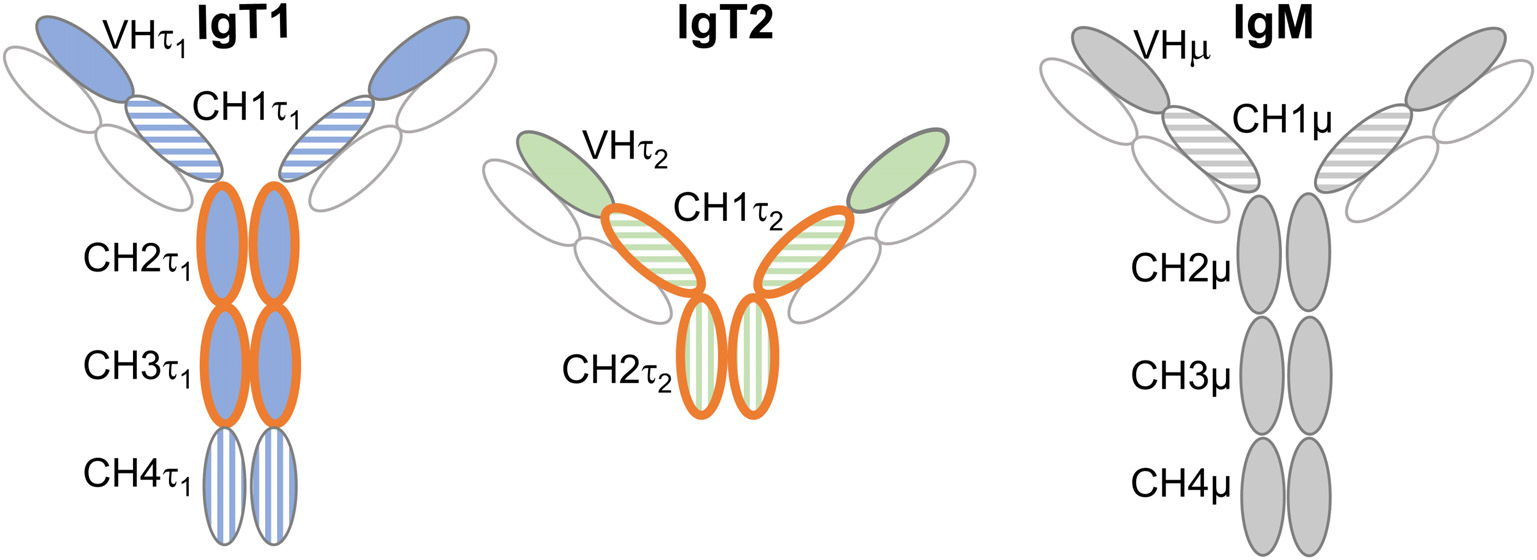
Domains used for production of anti-IgT1 and anti-IgT2 antibodies. Schematic representation of carp IgT1, IgT2 and IgM structure. Anti-IgT1 and anti-IgT2, were produced in chicken and rabbit, respectively using recombinant peptides corresponding to CH2τ_1_ and CH3τ_1_ of IgT1 or CH1τ_2_ and CH2τ_2_ of IgT2 (orange open circles). The horizontal and vertical striped circles indicate high amino acid similarity between CH1τ_1_, CH1τ_2_ and CH1µ, and between CH4τ_1,_ and CH2τ_2_ (see also **Supplementary Figures 1, 2**). Light chain is indicated in white circles. CH: constant heavy chain Ig domain.

Sequences spanning the confirmed CH2τ_1_-CH3τ_1_ of *IgHT1* and the CH1τ_2_-CH2τ_2_ of *IgHT2* (**Figure 1**) were codon optimized for bacterial expression and synthetized (BaseClear). Obtained sequences were subcloned in the expression plasmid pQE-30UA (QIAgen) between the *BamHI* and *HindIII* cloning sites, downstream of the sequence coding for six N-terminal histidines (6xHis-tag). Ligation products were cloned in M15 competent *Escherichia coli*, plated onto lysogeny broth (LB) agar plates supplemented with ampicillin (100µg/ml), kanamycin (25 μg/mL), and incubated overnight at 37°C. Positive clones were identified by colony PCR using vector-specific primers and the products were sequenced to verify correct orientation and reading frame. Selected positive clones were used for protein production as previously described (Piazzon et al., 2015). Briefly, bacteria were plated on LB-ampicillin-kanamycin plates and incubated overnight at 37°C. Bacterial suspension (40 mL) from an overnight culture was transferred to 1L TB medium (Bacto-tryptone 24g/L, Bacto-yeast extract 12g/L, NaCl 5g/L and Gycerol 4g/L) and incubated at 37°C until OD_600_ 0.6–0.8. Protein expression was induced by addition of 1 mM isopropyl β-d-thiogalactoside (IPTG) for 4 h at 37°C. Protein was purified from solubilized inclusion bodies under denaturing conditions using a Ni^2+^-NTA agarose beads (Qiagen) by gravity flow. Briefly, the column was washed with 5-column volumes of cold (4°C) 20 mM Tris-HCl, 500 mM NaCl, 6 M Guanidine-HCl, 25 mM imidazole, 1% (v/v) Triton X-100; 10-column volumes of cold 150 mM NaCl, 4 M Guanidine-HCl, 50 mM imidazole; Elution was performed with cold 150 mM NaCl, 4 M Guanidine-HCl, 250 mM imidazole. Purified proteins were immediately used for coupling to Aminolink Coupling Resin (Thermofisher) for subsequent affinity purification of antibodies. Alternatively, proteins were refolded by rapid 20x dilution in refolding buffer (1 mM EDTA, 2 mM DTT, 50 mM Tris-base, 500 mM NaCl, 5 mM GSSG, 1.25 M guanidine; pH 10.5), incubated overnight at 4°C and then dialyzed against 50 mM Tris-base, 500 mM NaCl (pH 10.5). The protein solution was centrifuged to remove any precipitate and concentrated again by binding on Ni^2+^-NTA agarose beads. Protein was eluted in 50 mM Tris-base, 250 mM Imidazole, pH 10.5. Protein specificity was assessed by Western blot analysis using an anti-penta-histidine antibody using the protocol described below. Purity of the eluted, refolded proteins was assessed by silver staining (Pierce, Thermo Fisher) prior to use for immunization of rabbit and chicken as described below.

### Antibody production and purification

Total IgG from pre-immune rabbits and total IgY from pre-immune chicken eggs, were screened by western blot analysis (described below) for absence of cross-reactivity to all recombinant proteins. Total rabbit IgG was purified using the Melon Gel IgG purification kit (Thermo Scientific) with gravity columns (Supelco), following the manufacturer’s protocol. Total IgY were purified from the pooled egg yolk by the ‘‘water dilution method’’ followed by ammonium sulphate precipitation as previously described (Hansen et al., 1998). Protein concentrations of the purified total IgY and total IgG fractions were measured at 280 nm using a Nanodrop-1000 (Thermo Scientific) and were stored either at -20°C or at 4°C after addition of 0.01% sodium azide. Polyclonal rabbit serum anti-IgT2 was produced by immunization of rabbits with purified refolded bacterial recombinant IgT2 (1 x 200 μg followed by 3 x 100 μg) according to a 3-month standard protocol (Naxo, Estonia). Polyclonal chicken IgY anti-IgT1 was produced in house by immunization of chicken with purified recombinant IgT1 (2 x 100 μg of protein using Supecol as adjuvant (#7925000, WBVR Lelystad, The Netherlands), with four weeks interval). Yolk from eggs containing highest titers of anti-IgT1 were selected by ELISA using the recombinant protein as antigen (described below), and pooled. Rabbit IgG anti-IgT2 were affinity purified from rabbit serum using an AminoLink coupling resin (Thermo Scientific) containing 2 mg of purified recombinant IgT2 according to the manufacturer’s protocol with slight modification. Briefly, protein was coupled to the resin using 150 mM NaCl, 4 M Guanidine-HCl, 250 mM imidazole as coupling buffer. Column was used immediately or stored at 4°C in PBS containing 0.01% sodium azide.

### Indirect ELISA

For indirect ELISA, high-binding plates (Greiner Bio-One) were coated overnight at 4°C with serial dilutions of recombinant IgT1 or IgT2 in carbonate/bicarbonate buffer (pH 9.6). Plates were blocked for 1 h at room temperature (RT) with 3% (w/v) bovine serum albumin (BSA) in phosphate-buffered saline (PBS) with 0.05% Tween (PBST). Plates were then incubated with serial dilutions of the chicken anti-IgT1 and rabbit anti-IgT2 primary antibody in 3% BSA-PBST and subsequently with HRP-conjugated rabbit-anti-chicken (1:4000; ImmunoResearch Laboratories) or HRP-conjugated goat-anti-rabbit (1:2000, Dako). The reaction was developed with 2,2’-azino-bis (3-ethylbenzothiazoline-6-sulphonic acid) (ABTS; Roche) and OD_405nm_ was measured using a Filtermax F5 multi-mode microplate reader (Molecular Devices).

### Collection of serum, gills-, gut- and skin-mucus and mucus-associated bacteria

Carp were euthanized with an overdose (0.6 g/L) of Tricaine Methane Sulfonate (TMS, Crescent Research Chemicals) buffered with 1.2 g/L NaHCO3. For serum collection, blood was collected by puncturing the caudal vein and allowed to clot at 4°C for >2h. Samples were centrifuged at 800*xg* for 10 minutes at 4°C and the supernatant was collected. Serum from more than 10 individuals was heat inactivated at 56°C for 30 minutes before being pooled, aliquoted and stored at -20°C until further use. Gills mucus and the associated bacteria were isolated as described previously in rainbow trout (Xu et al., 2016) with slight modifications: gills were perfused with Cortland solution (124.1 mM NaCl, 5.1 mM KCl, 2.9 mM Na2HPO4, 1.9 mM MgSO4, 1.4 mM CaCl2, 11.9 mM NaHCO3, 5.5 mM D+-glucose, 30 IU Heparin, 1% Penicillin-streptomycin, Osmolarity (270-290 mOsm/kg), pH 7.5), arches were excised and washed three times in PBS to remove any remaining blood. Gill arches were transferred to PBS containing 1x protease inhibitor cocktail (Sigma, P8849), 1 mM Pefabloc SC plus with PSC protector (Sigma, #11873601001) at 1.3 ml buffer/gram of tissue and incubated for 12 h at 4 °C in the dark, with gentle shaking. The mucus-containing suspension was collected and passed through 100 μm cell strainer. It was then transferred to a low protein-binding Eppendorf tubes and centrifuged at 1000x*g* for 10 min at 4°C to separate cells. After centrifugation, the mucus-containing supernatant was collected and centrifuged at 10,000x*g* for 10 min to separate the mucus and pellet the mucus-associated bacteria. The resulting cell-free and bacteria-free supernatant were collected and filtered through a 0.45 μm filter (Millipore). The protein concentration was measured by NanoPhotometer N60 (Implen) at 280 nm. The pelleted bacteria were washed three times with PBS. To avoid protein degradation, bacteria and mucus were boiled in protein sample buffer containing β-mercaptoethanol, aliquoted, and stored at -20°C for further analysis on western blot.

To collect skin mucus and its associated bacteria, carp were euthanized as described above. TMS was removed from the skin by rinsing the carp in clean tank water. Fish were bled and mucus was collected from the left flank by gently scraping the skin with a cell scraper being careful to not come near the bleeding site. The mucus was transferred to a low protein-binding tube and diluted 1:6 with 1x protease inhibitor cocktail (SIGMa, P8849). The sample was vortexed, and mucus and bacteria were separated by sequential centrifugation as described above allowing first separation of any scale or skin cells followed by separation of skin bacteria and skin mucus.

To collect gut mucus and its associated bacteria, the gut was closed with Kocher-Pean clamps on both sides and excised. Fat or other tissue were removed, and the gut was washed with cold PBS. Then, about 2 ml of PBS containing 1x protease inhibitor cocktail, 0.5 mg/ml soybean trypsin inhibitor (Roche, #10109886001), 1 mM Pefabloc SC plus with PSC protector and 0.5% BSA, pH 7) were added into the gut tube and subsequently collected. Mucus and bacteria were isolated by sequential centrifugation as described above and stored at -20°C until further processing on western blot.

### Mono-dimensional electrophoresis and western blot analysis

To detect recombinant IgT1 and IgT2 or soluble carp IgT1, IgT2 and IgM, samples were resolved on 4–20% precast SDS-PAGE (BIO-RAD), under reducing conditions. Samples with the following total protein concentrations were loaded per lane: purified recombinant proteins (0.1 μg), naïve pooled carp serum (20 µg), gills-mucus (60 µg) skin and gut mucus (120 µg), gills bacteria (equivalent to 1 ml of gills mucus), skin and gut bacteria (equivalent to 100 µl of mucus). The gel was run at 120 V for 1 hour. After separation, proteins were transferred to nitrocellulose membranes (Protean, Thermo Scientific), using semi-dry transfer cell (BIO-RAD) and blocked for 1h with 5% (w/v) non-fat dry milk (NFDM, Elk). All subsequent steps were performed at room temperature (RT), unless stated otherwise. Membranes were incubated overnight at 4°C with the following concentrations of primary antibodies diluted in blocking buffer: anti-penta-histidine mouse antibody (1:5000, QIAgen, 34660), 10 μg/ml of total chicken IgY anti-IgT1, 2.5 μg/ml of affinity purified rabbit IgG anti-IgT2 and 1:100 dilution of the hybridoma supernatant containing the monoclonal mouse IgG1 anti-carp IgM (WCI12, 2 μg/ml) (37). After washing, membranes were incubated with either HRP-conjugated rabbit anti-chicken (1:4000, ImmunoResearch Laboratories, 303-035-003) or HRP-conjugated goat-anti-rabbit (1:2000, Dako, P044801-2) or HRP-conjugated goat-anti-mouse (1:2000, Dako, P044701-2) diluted in 10% (w/v) NFDM. Development was performed with Pierce ECL Western blotting substrate (Thermo Fisher, 34577) and chemiluminescence signal was visualized using a ChemiDoc XRS and corresponding software (BioRad). Alternatively, membranes were incubated with either alexa-680-conjugated goat anti-mouse IgG (Abcam, Ab175775) and IRDye-800-conjugated donkey anti-chicken-IgY (Licor, 925-32218) or with RDye-800-conjugated goat anti-rabbit (Licor, 926-32211) and alexa-647-conjugated goat anti-mouse IgG (Invitrogen, A21235) or alexa-680-conjugated goat anti-mouse IgG. Where RDye-800-conjugated goat anti-rabbit IgG was used in combination with alexa-680-conjugated goat anti-mouse IgG, cross-reactivity of the latter secondary antibody to the rabbit primary antibody was observed. In this case, membranes were first developed to visualize IgM, followed by labelling for IgT2. After secondary antibody incubation and washings, images were captured using the infrared fluorescence imager Odyssey (LI-COR) and data were analysed using Image Studio Lite software (LI-COR).

### Two-dimensional gel electrophoresis

Naïve pooled carp serum (40 µg of total protein) or skin bacteria collected from 300 µl of skin mucus, were boiled under reducing conditions and proteins were precipitated by acetone precipitation method (Lyons, 2003), to remove salts. Briefly, four volumes of cold acetone (-20°C) were added to one volume of protein sample, vortexed and incubated for 1 hour at -20°C. Next, the samples were centrifuged for 10 minutes at 13.000 xg. Supernatants were discarded and the tubes were left open for 20 minutes to allow residual acetone to evaporate. Subsequently, each pellet was dissolved in 125 µl of rehydration buffer pH 8.8 (8 M Urea, 2% CHAPS, 20 mM DTT, 0.5% IPG buffer pH 3-11 (GE Healthcare, GE17-6001-12), and trace amount of bromophenol blue). Seven cm Immobilized pH gradient strip (IPG strip) pH 3-10 (GE Healthcare Bioscience, GE17-6001-12) was incubated with 125 µl of protein-containing rehydration buffer in a 7 cm rehydration tray. All subsequent steps were performed at RT, unless stated otherwise. After 1 hour, each strip was covered with 1 ml of mineral oil to avoid evaporation and incubated overnight. The next day, the IPG strips were subjected to isoelectric focusing (IEF), using protean IEF cell (BIO-RAD). Samples were focused at 20°C as follows: 300 V 0.2 kVhr linear gradient; 1000 V 0.3 kVh rapid gradient; 5000 V 4kVh rapid gradient; 5000 V 2kVhr linear gradient. After the first dimension of electrophoresis, the IPG strips were equilibrated in the equilibration buffer pH 8.8 (6 M Urea, 50 mM Tris, 2% SDS, 60 mM DTT, 30% glycerol, and trace amount of bromophenol blue). To run the second-dimension electrophoresis, IPG strips were layered on 4-20% precast gel, 7 cm IPG/prep well (Bio-Rad, 4561091) and a dual-color protein marker (BIO-RAD) was loaded beside the positive end of the strip. The gel was run at 100 V for 1.5 hour. Finally, proteins were transferred to nitrocellulose membranes and western blot was conducted according to the procedures mentioned above.

### Antibody specificity test: peptide blocking assay

Total chicken IgY anti-IgT1 (10 µg/ml) and affinity purified rabbit IgG anti-IgT2 (2.5 µg/ml) were pre-incubated with 10-fold molar excess of the respective immunization peptides. The reaction was performed in tris-buffered saline (TBS) containing 5% (w/v) non-fat dry milk (NFDM, Elk) and 0.05% (v/v) tween (TBST) and incubated one hour at room temperature. Subsequently, western blot analysis was performed as described above.

### De-glycosylation of skin-mucus proteins

Skin-mucus proteins were boiled under reducing conditions and 200 µg were precipitated by acetone precipitation to remove salts. Protein pellets were dissolved in 18 µl of 50 mM ammonium bicarbonate buffer (pH 7.8) and 20 U of PNGase (Promega, V4831) was added to the appropriate tube. The samples were mixed and incubated at 37°C for 3 hours. Loading buffer was added to the samples, boiled, and loaded on 4–20% precast SDS-PAGE. Western blot was performed as described above.

### Samples preparation and mass spectrometry

Because anti-IgT1 and anti-IgT2 do not recognize their respective proteins under native conditions, and because immunoprecipitation is not possible in this case, protein bands corresponding to the putative IgT1 and IgT2 were excised from acrylamide gel after 2D separation and subjected to Mass Spectrometry (MS). To this end, samples from skin bacteria isolated and cleaned up as described above were used for MS; in this way only carp proteins bound to skin bacteria would be present in the sample, thereby enriching samples for IgT1 and IgT2. In parallel, protein bands equivalent to putative carp IgT1 excised from serum samples after 2D separation were also used. Samples were treated and separated by 2D gel electrophoresis as described above. 2D gel were performed in duplicate; one gel was used to visualize IgT1 or IgT2 by western blot and the other gel was stained with Flamingo™ Fluorescent Protein Gel Stain (BIO-RAD, #1610491) and used to excise bands of proteins of interest. Samples were prepared for MS following in-gel digestion. Briefly, gel slices were washed three times with distilled water, followed by incubation with 15 mM DDT (SIGMA) for 30 min at 45 °C on a shaker to reduce proteins. Gel slices were washed twice with distilled water to remove DTT. Unless stated otherwise, all the chemicals were diluted in 50 mM ammonium bicarbonate solution (SIGMA). Next, to alkylate proteins, gel slices were incubated with 20 mM acrylamide for 30 min at room temperature in the dark and washed three times with distilled water. After alkylation, tryptic digestion was performed overnight at room temperature on a shaker using 5 µg/ml sequencing grade trypsin (Roche, 11047841001). Peptides were collected after overnight trypsin digestion and loaded on the double C8-Filter-Tip (200 ul tips). The C8-Filter-Tip were placed in a low protein-binding tube and eluted. Fifteen µl of 50% acetonitrile (50% acetonitrile, 50% water and 0.1% formic acid) was added to the filter and eluted again in the same low protein-binding tube. The acetonitrile volume in the eluates was reduced to less than 10 µl by centrifugation using SpeedVac. The sample volume was adjusted to 25 µl using 0.1% formic acid, samples were then measured by LC-MS/MS and analysed as described previously (42).

### Immunofluorescence staining

Carp were euthanized, blood was collected from the caudal vein, and gills were perfused as described above. Gills, head kidney, spleen and gut were aseptically removed, embedded in Tissue-Tek^®^ O.C.T. Compound (Sakura, 4583), frozen in liquid nitrogen and stored at -80. Tissues were sectioned at 5 μm thickness using a Leica CM3050S Cryostat Microtome (Leica) and mounted on polylysine-coated slides. Cryosections were air-dried for 1 hour at room temperature and fixed for 15 min at 4 °C with 4% paraformaldehyde (PFA). All subsequent steps were performed at room temperature (RT), unless stated otherwise. After washing three times in PBS-0.1% Triton (PBS-T), slides were blocked with 10% normal goat serum (DAKO, X 0907) in PBS with 0.05% tween-80 for 30 minutes. Immunofluorescence staining was performed by incubating the slides overnight at 4°C with the following primary antibodies diluted in 1% normal goat serum in PBS-0.05% tween-80: total chicken IgY anti-IgT1 (15 µg/ml), affinity purified rabbit IgG anti-IgT2 (5 µg/ml), hybridoma supernatant containing the monoclonal mouse IgG anti-carp IgM (WCI12, 1:100, 2μg/ml). After incubation with the primary antibodies, slides were washed and incubated for 1 hour with the respective secondary antibodies diluted in 1% normal goat serum in PBS-0.05% tween-80 as follows: Alexa 647-conjugated goat anti-chicken IgY (Invitrogen, A21449), FITC-conjugated goat anti-mouse IgG (Abcam, Ab6785), 4 µg/ml from each, and TRITC-conjugated swine anti-rabbit IgG, 1:100 (DAKO, R0156). To ensure specificity of the reactions, each secondary antibody was incubated with the irrelevant primary antibodies and in all four tissues of interest, each secondary antibody reacted only to the corresponding primary antibody (**Supplementary Figures 3** and **4**). After three washing steps, slides were incubated with 1x DAPI solution (4’,6-Diamidino-2-Phenylindole, Dihydrochloride, Invitrogen, D1306). Finally, slides were mounted with fluorescence mounting medium (Vectashield, Vector laboratories) and images were acquired using DM6b upright fluorescence microscope (Leica). Images were analysed using ImageJ software (National Institute of Health, version 1.53c).

### Infection with *Trypanoplasma borreli* and sample collection

Carp (9-months-old) were intra-peritoneally (i.p.) infected with 1 x 10^4^ *T. borreli* parasites (43), as described before (44). At 3 weeks after infection, carp were euthanized, and blood was isolated from the caudal vein for subsequent serum isolation; in parallel spleen from control (non-infected) and infected carp were isolated and immediately embedded in Tissue-Tek^®^ O.C.T. Compound (Sakura, 4583) prior to freezing in liquid nitrogen until processing for immunofluorescence. *T. borreli* parasites were isolated from heparinized blood as described before (44) and cultured in HML medium (45% HBSS, 22.5% MEM, 22.5% L15 (all from Gibco) and 10% distilled water) supplemented with 3% pooled carp serum, 1% Penicillin-streptomycin, and 1% L-glutamine.

### *T. borreli-*specific immunoglobulins binding

To evaluate the development of *T. borreli*-specific antibodies in the serum of infected carp, parasites were kept in culture for at least two days after isolation to remove any surface-bound carp serum protein and to allow their degradation. We previously showed that motile *T. borreli* can effectively remove any surface-bound protein, including immunoglobulins, within 2 minutes (45); thus, to prevent newly bound immunoglobulins from being internalized by motile parasites, cultured parasites were immediately fixed with 4% PFA in PBS for 10 minutes at 4°C, and washed three times with cold PBS prior to blocking in PBS-0.5% BSA for 45 minutes at 4°C. Parasites were washed in PBS and incubated for 1 hour at 4°C in serum collected 3 weeks post-*T. borreli* infection, or in serum from non-infected controls diluted in PBS (1:1, 1:10 or 1:50 dilutions). Parasites were pelleted and the equivalent of 40,000 parasites from each serum dilution were resolved on SDS-PAGE under reducing conditions. Western blot analysis was performed as described above.

## Results

### Validation of *IgHT1* and *IgHT2* transcripts for European common carp

Previous studies in East-Asian common carp reported the presence of two *IgHT* subclasses that were designated *IgHZ1* and *IgHZ2* (9, 15). We took advantage of the availability of the genome (16) and full-body transcriptome (17) of European common carp to verify the structure of the *IgHT* transcripts and corresponding protein models prior to recombinant protein production. For clarity, in the current manuscript we will refer to the European common carp sequences as *IgHT1* and *IgHT2* (instead of *IgHZ1* or *IgHZ2*) to comply with the original and newly proposed nomenclature (3, 41); however we keep the IgZ nomenclature when discussing previous work made from Asian carps as used in the corresponding reports. Using the East-Asian common carp *IgHZ1* (Acc No: BAJ41037) and *IgHZ2* (Acc No: BAJ41038) sequences reported previously as reference for a BLAST search, two carp *IgHT* genes were retrieved on two separate scaffolds and were designated *IgHT1* (scaffold 44206) and *IgHT2* (scaffold 46295) (**Supplementary Figure 2A**) based on their highest similarity to the corresponding East-Asian common carp sequence. For *IgHT2* we retrieved two exons encoding two CHτ domains translating into a protein of 220 aa with an overall 97% amino acids (aa) identity to the previously published *IgHZ2* sequence (**Supplementary Figures 2B, D**). For *IgHT1*, on scaffold 44206, two out of the four identified exons encoded CHτ domains with high sequence similarity to CH3_ζ1_ and CH4_ζ1_ of the published *IgHZ1* sequence; the other two exons had clear homology to the CH1_ζ1_ and CH2_ζ1_ of *IgHZ1* but shared only 37% and 52% amino acid identity with these sequences, respectively. During the course of this study, another East-Asian carp *IgHZ* sequence (named *IgHZ3*, Acc No: QHW04710) (46) was reported, with an overall 90% identity to our *IgHT1* sequence, including 84% and 90% identity with CH1τ_1_ and CH2τ_1_ domains, respectively (**Supplementary Figures 2B, C**). We also realized that the original manuscript describing the East-Asian common carp *IgHZ1* sequence reported a gene model based only on predictions from genomic sequences. Whether the predicted exons were all transcribed and spliced consecutively, and thus contributed to the expressed mRNA, was never reported. In the current study, full-length sequence of European carp *IgHT1* were amplified by RT-PCR analysis using forward primers annealing at the 5’ end of various VH domains and reverse primers spanning the CH2τ_1_ or CH4τ_1_ domain. Sequences of these PCR products confirmed that soluble European carp *IgHT1* contains four CH domains (Acc No PQ441757) and that sequences of the CH1τ_1_ and CH2τ_1_ domain matched the newly reported *IgHZ3* but had low sequence similarity with CH1_ζ1_ and CH2_ζ1_ sequences of the East-Asian carp *IgHZ1*. Attempts to amplify the full length *IgHZ1* sequence using primers located in CH1_ζ1_ and CH4_ζ1_ reported in (9) yielded no PCR product when total RNA isolated from head kidney or spleen was used as template (data not shown). We conclude that the *IgHZ1* mRNA model reported in Ryo et al. (9) is not expressed in the European carp we used. As it was not found by Zhang et al. (46) in Asian carp, it may in fact correspond to an incorrect prediction. We will therefore name IgHT1 the heavy chain with four CH domains encoded by our 1247 bp mRNA amplified from European common carp (**Supplementary Figures 2B, C**). Comparison of European carp *IgHT1* and *IgHT2* sequences (**Supplementary Figure 2E**) with common carp *IgHM* sequences (**Supplementary Figure 1**) showed a high percentage of identity of their CH1 domains (72% between *IgHT1* and *IgHT2*, 79% between *IgHT1* and *IgHM* and 70% between *IgHT2* and *IgHM*). Furthermore, European common carp CH4_τ1_ and CH2_τ2_ domains were highly similar (70% identity), but more distant from the carp IgM CH4_μ_ domain (20-25% identity between both *IgHT* sequences and *IgHM* CH4_μ_ domain). Altogether we report two *IgHT* sequences, *IgHT2* almost identical to the previously reported *IgHZ2* sequence, and *IgHT1*, differing from the original *IgHZ1* especially at the level of the CH1τ_1_ and CH2τ_1_ domains.

### Specificity of antibodies against recombinant IgT1 and IgT2

Polyclonal antibodies were raised against carp IgT1 and IgT2 by immunizing chicken and rabbits (respectively) with purified recombinant proteins corresponding to the CH2τ_1_ and CH3τ_1_ domain of IgT1, and to the CH1τ_2_ and CH2τ_2_ domain of IgT2 (**Figure 1** and **Supplementary Figures 5A-E**), respectively. The choice for the IgT1 domains was based on the low similarity to those of IgM or IgT2. For IgT2, our choice was a compromise since the CH1τ_2_ domain showed high similarity to the CH1 domain of both IgM and IgT1, and the CH2τ_2_ domain showed similarity to the IgT1 CH4τ_1_ (**Supplementary Figures 1**, **2**). Total chicken-IgY anti-IgT1 and total rabbit-IgG anti-IgT2, were preliminary tested by western blot analysis for their ability to specifically recognize the recombinant proteins used for immunization, and to not recognize the other recombinant protein (**Supplementary Figure 5F**). In parallel, the monoclonal antibody against carp IgM was also tested for its potential cross-reactivity to recombinant IgT2 (**Supplementary Figure 5G**). Anti-IgT1 antibodies specifically recognized recombinant IgT1 domains (25 kDa), and anti-IgT2 antibodies reacted to the recombinant IgT2 domains (25 kDa). No cross-reactivity of anti-IgM to recombinant IgT2 was observed. After assessing the specificity of the antibodies by Western blot analysis, we determined the sensitivity and specificity of the antibodies by ELISA. Both antibodies could reliably recognize the autologous recombinant protein at concentrations lower than 30 ng/ml (**Supplementary Figure 5H**). Anti-IgT1 did not react against recombinant IgT2, and vice versa (**Supplementary Figure 5H**, dotted lines). Altogether, antibodies raised against European common carp IgT1 and IgT2 showed consistent reactivity and specificity to the recombinant protein used for immunization.

### IgT1 and IgM are detected in serum, mucus and mucus-associated bacteria

**Figure 2:**
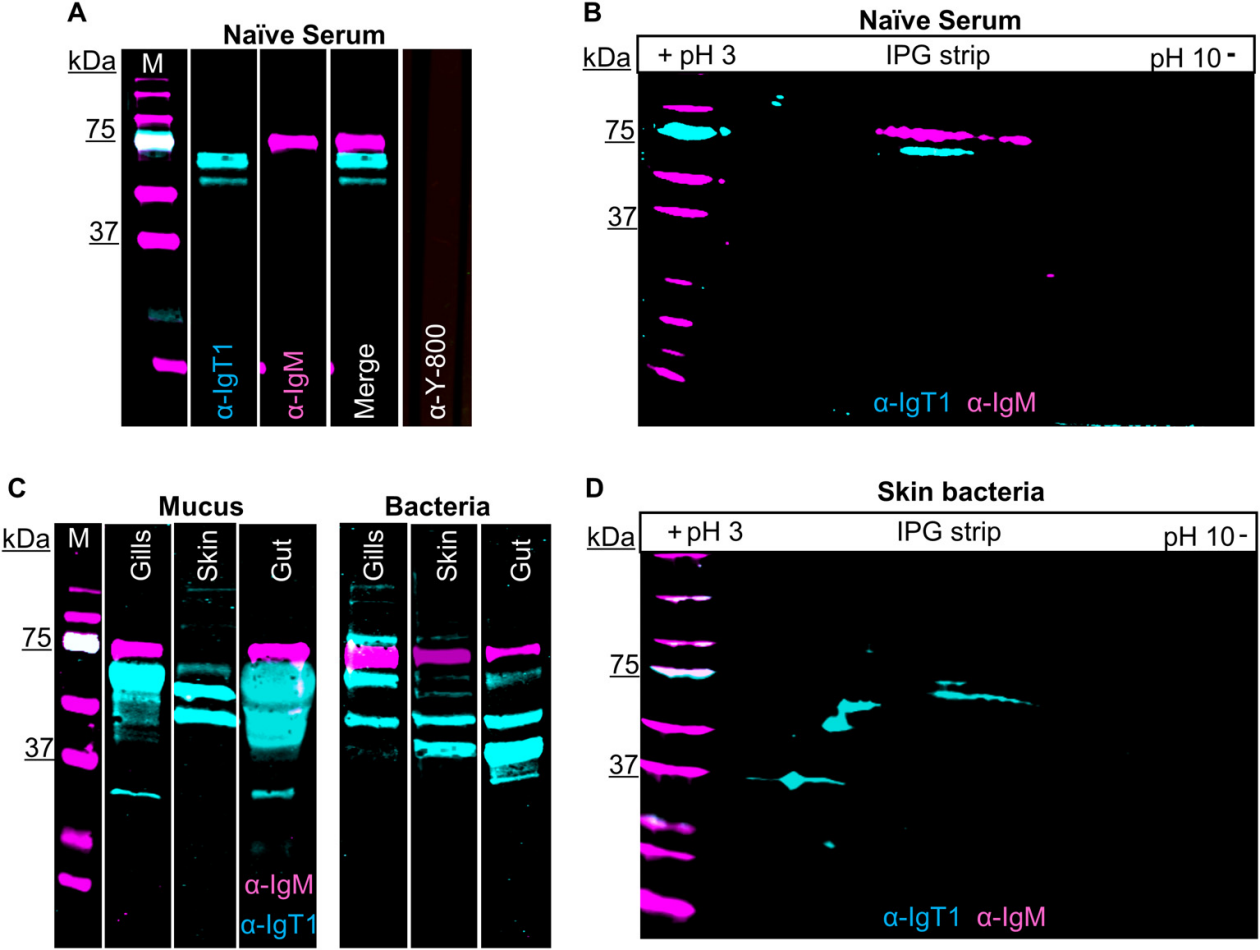
Presence of carp IgT1 and IgM in serum, mucus and mucus-associated bacteria. Detection of IgT1 and IgM in naïve serum, mucus and mucus-associated bacteria by Western blot analysis. M indicates the molecular weight (Mw) marker in kilodaltons (kDa). Samples were resolved on an SDS-PAGE under reducing condition **(A, C)** or were first separated according to their isoelectric points (pI), followed by separation according to their molecular weight by 2D electrophoresis **(B, D)**. Chicken anti-IgT1 (α-IgT1, 10 µg/ml) and mouse-IgG-anti-IgM (α-IgM, 2 μg/ml) primary antibodies and IRDye800-conjugated donkey anti-chicken (0.1 µg/ml, pseudo-colored cyan) and alexa-680-conjugated goat anti-mouse-IgG (0.1 µg/ml, pseudo-colored magenta) secondary antibodies were used for detection. Images were captured with infrared florescence imager (Odyssey). In general, an intensity of 5 was used to capture fluorescence signals, but in **C,** intensity of 4.5 was used to capture IRDye800-conjugated donkey anti-chicken and intensity of 6 was used to capture alexa-680-conjugated goat anti-mouse-IgG due to the strength of the IgT1 signal. **(A, B)** Detection of IgT1 and IgM in naïve serum. **(A)** 20 µg/lane; **(B)** 40 µg. Naïve serum incubated with only IRDye800-conjugated donkey anti-chicken as secondary antibody served as control (**A**, last lane). A 61 kDa protein corresponding to the predicted Mw of IgT1 (cyan) and a 75 kDa protein band corresponding to IgM (magenta) are visible. **(C)** Detection of IgT1 and IgM in mucus isolated from gills, skin and gut (left) and in the respective mucus-associate bacteria (right). Samples loaded on gel: 60 µg total protein from gills’ mucus, 120 µg total protein from skin and gut mucus; gills bacteria extracted from 1 ml of gills mucus; skin and gut bacteria extracted from 100 µl of mucus. **(D)** Detection of IgT1 and IgM after 2D gel electrophoresis of skin mucus-associated bacteria extracted from 300 µl of skin mucus.

We then examined the presence of IgT1 and IgM in serum, mucus, and mucus-associated bacteria. In serum, anti-IgT1 specifically reacted to a protein of about 61 kDa, which corresponds to the theoretical molecular weight of the heavy chain of soluble carp IgT1 (**Figure 2A**, cyan). Similarly, anti-IgM specifically recognized a 75 kDa protein corresponding to the heavy chain of soluble carp IgM (**Figure 2A**, magenta). Next, we examined the presence of IgT1 and IgM in serum using 2D gel electrophoresis followed by western blot analysis. Also here, anti-IgT1 and anti-IgM recognized two peptides with distinct molecular weight, and isoelectric point (pI) of around 6.5 (**Figure 2B**). We further investigated the presence of IgT1 and IgM proteins in mucus from gills, skin, gut and their associated bacteria. IgT1 was detected in mucus from gills, skin and gut as well as from the associated bacteria. Similarly, IgM could be detected in mucus and mucus-associated bacteria, although in skin mucus, IgM concentration appeared very low, and the signal was visible only after overexposure (**Figure 2C**). In mucus and mucus-associated bacteria, beside the 61kDa peptide, anti-IgT1 recognized peptides of multiple molecular weights (around 79 kDa, 50 kDa and 37 kDa) (**Figure 2C, D**). The peptides with a molecular weight <61 kDa could be degradation products of IgT1 due to the presence of proteases in the mucus; the proteins with a higher molecular weight could be highly glycosylated forms of IgT1. To confirm the specificity of the bands recognized by the anti-IgT1, the antibody was blocked by pre-incubation with the immunization peptide prior to western blot analysis. In this experiment, where skin mucus was used, the IgT1 signal disappeared when the antibody was blocked with the immunization peptide, (**Supplementary Figure 6A**). To further confirm the specificity of the bands recognized by the anti-IgT1 antibody, MS analysis was conducted on gel slices excised after 2D gel electrophoresis of skin mucus-associated bacteria and of serum pools from naïve carp (**Supplementary Figure 6B**). In total, five distinct peptides were identified that had 100% match with the IgT1 sequence of the European common carp (**Supplementary Figure 6C**): in the samples derived from skin mucus-associated bacteria, all peptides were detected in the sample corresponding to the 61 kDa protein band of IgT1, two peptides were detected in the sample corresponding to the 50 kDa protein band and no peptide matching IgT1 was detected in the sample corresponding to the 37 kDa protein band. One peptide was detected in the sample corresponding to the 61 kDa protein band extracted from naïve serum. Overall, our data reveal the presence of IgT1 and IgM in serum, mucus and mucus-associated bacteria. In addition, the specificity of the anti-IgT1 antibody to the native IgT1 protein was confirmed by peptide blocking assay and MS analysis.

### IgT2 is present in mucus and mucus-associated bacteria, but not in serum

**Figure 3:**
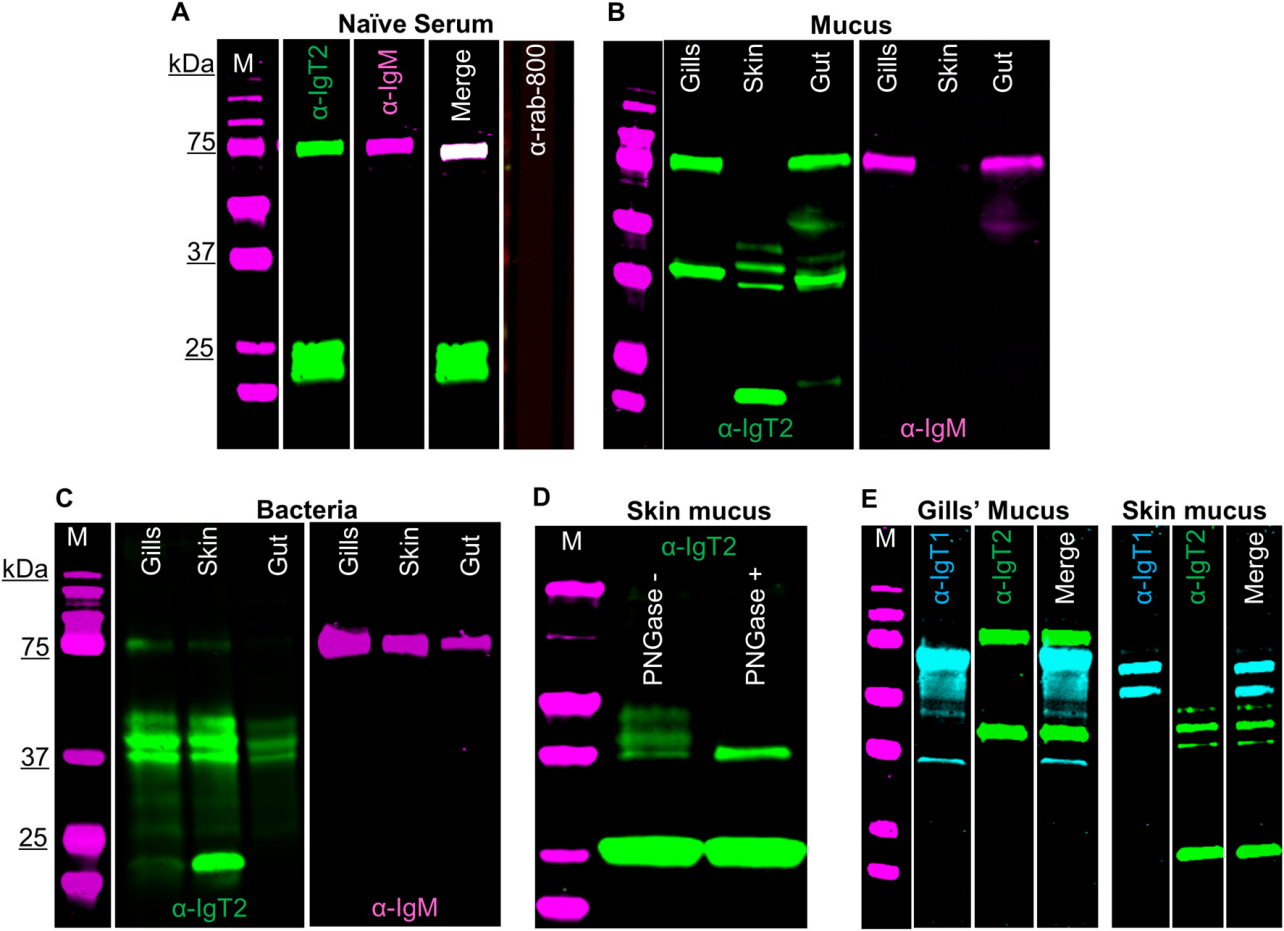
Detection of carp IgT2 and IgM in serum, mucus and mucus-associated bacteria. Western blot analysis to detect IgT2 and IgM in naïve pooled-carp serum (naïve serum) **(A)**, in mucus **(B)** and in mucus-associated bacteria **(C)**. Protein concentrations were as follows: naïve pooled-carp serum (naïve serum, 20 µg of total protein), gills’ mucus (60 µg of protein), skin and gut mucus (120 µg of protein), gills’ bacteria isolated from 1 ml of gills’ mucus, skin and gut bacteria isolated from 100 µl of mucus. Samples were resolved on an SDS-PAGE gel under reducing condition and western blot was performed using rabbit anti-IgT2 (2.5 µg/ml, α-IgT2) and mouse-anti-IgM (2 μg/ml, α-IgM), followed by incubation with IRDye800-conjugated goat anti-rabbit (0.1 µg/ml, green) and alexa-647-conjugated goat anti-mouse IgG **(A)** or alexa-680-conjugated goat anti-mouse-IgG **(B, C)**, (0.2 µg/ml, pseudo-colored magenta). A sample incubated with only IRDye-800-conjugated goat anti-rabbit secondary antibody served as control (**A, last lane**). A 39 kDa protein corresponding to the predicted Mw of IgT2 (green) and/or a 75 kDa protein corresponding to IgM (magenta) as well as a 22 kDa protein (green) corresponding to an unknown protein recognized by the anti-IgT2 are visible. **(D)** Western blot analysis of skin mucus (200 µg of protein), denatured, then untreated or treated with 20 U PNGase (de-glycosylation enzyme), prior to resolving in SDS-PAGE. After de-glycosylation of the sample, only a single band of 39 kDa, corresponding to the expected molecular weight of IgT2, could be detected on western blot. **(E)** Detection of IgT1 and IgT2 in gills and skin mucus. The same concentration of proteins described in B were resolved in SDS-PAGE under reducing conditions. IgT1 and IgT2 were detected using chicken anti-IgT1 (10 µg/ml) and rabbit anti-IgT2 primary antibodies, followed by IRDye-800-conjugated donkey anti-chicken (0.1 µg/ml, pseudo-colored cyan) and IRDye-700-conjugated goat anti-rabbit (0.1 µg/ml, pseudo-colored green). Images were acquired with infrared florescence imager (Odyssey). In general, an intensity of 5 was used to capture fluorescence signals, but in B and C, an intensity of 4.5 was used to capture IRDye800-conjugated goat anti-rabbit (green signal) and an intensity of 6 was used to capture alexa-680-conjugated goat anti-mouse-IgG (magenta signal). M: molecular weight marker; kDa: kilodaltons.

Using a similar strategy as the one described for the anti-IgT1 antibody, we examined the presence of IgT2 in naïve serum, mucus and mucus-associated bacteria. No reactivity to a protein of the predicted theoretical molecular weight of the heavy chain of soluble IgT2 (39 kDa) was observed in naïve serum. However, anti-IgT2 showed cross-reactivity to the 75 kDa protein corresponding to the heavy chain of soluble carp IgM (**Figure 3A**). The cross-reactivity was likely due to the high similarity between CH1τ_2_ and CH1µ. Anti-IgT2 antibodies also recognized an unknown 22 kDa protein not recognized by the anti-IgM antibody (**Figure 3A**).

Next, mucus and mucus-associated bacteria were investigated for the presence of IgT2. Differently from what was observed in serum, peptides around 39 kDa, corresponding to the predicted apparent molecular weight of the heavy chain of soluble IgT2, were readily detected in mucus from gills, gut and skin as well as in the respective mucus-associated bacteria (**Figures 3B, C**). In line with the results obtained from serum, cross-reactivity of anti-IgT2 to the 75 kDa heavy chain of the IgM protein was observed also in mucus samples and, to a lesser extent, in samples from mucus-associated bacteria (**Figures 3B, C**). The lower reactivity to IgM is likely due to the lower abundance of IgM in mucus-associated bacteria compared to IgT2 as suggested by the fact that in **Figures 3B, C** the intensity of the magenta channel is higher (6) than for the green channel (4.5). This also explains the absence of IgM in skin mucus. In skin mucus, IgM had a very low abundance and IgM signal was only visible when the signal was over-exposed (**Figure 3B**, third and sixth lane). The unknown 22 KDa protein recognized by anti-IgT2 in naïve serum, was also detected in mucus and mucus-associated bacteria from all compartments except in gill mucus and gut bacteria. In all mucus samples and their associated bacteria, except in gill mucus, at least three bands around the expected molecular weight of 39 kDa were observed. We hypothesized that at these mucosal sites IgT2 could be present in glycosylated form. Indeed, after treatment of skin mucus with the de-glycosylating enzyme (PNGase), only a single band around 39 kDa was detected (**Figure 3D**). To further confirm the specificity of the anti-IgT2 and anti-IgT1 antibodies, we combined IgT1 and IgT2 detection in one blot using gills and skin mucus (**Figure 3E**). The results indicated that the anti-IgT2 specifically reacted to IgT2 and not to IgT1 and vice versa. An additional test for specificity was performed by blocking the reactivity of anti-IgT2 with recombinant IgT2 prior to western blot analysis (**Supplementary Figure 7A**). These results confirmed specificity by showing absence of IgT2 signal when the antibody was blocked with the recombinant peptide.

To investigate the nature of the unknown 22 kDa protein, MS analysis was performed on gel slices excised after 2D gel electrophoresis of skin mucus-associated bacteria. In parallel, western blot was performed on a duplicate 2D gel using anti-IgT2. Results showed the 39 kDa protein of IgT2 around the predicted isoelectric point (pH 8.6), while the 22 kDa protein was observed around pH 6 (**Supplementary Figure 7B**). MS data confirmed the presence of seven peptides with a 100% match to IgT2 (scf46259) in samples extracted from gel slices corresponding to the 39 kDa protein (**Supplementary Figure 7C**), whereas no peptide matching IgT2 was identified in the sample corresponding to the 22 kDa protein band, suggesting that this is not an IgT2 degradation product. Among the most abundant proteins identified in this sample and matching the expected molecular weight, we find proteins related to C1q-like complement molecules and several unknown proteins (data not shown); among the least abundant, yet present in the sample, are three peptides matching IgM two of which are located in the CH3µ domain and one in the CH4µ. Since these CHµ domains share low protein similarity to IgT2, and the retrieved peptides were not among the most abundant, we can also exclude that the 22 kDa protein is a degradation product of IgM. Definite identification of the nature of the 22 kDa protein thus awaits further characterization. Taken together, we confirmed the specificity of the anti-IgT2 to the native IgT2 protein by western blot analysis, peptide blocking assay and MS analysis, although cross-reactivity to soluble IgM and to an unknown 22 kDa protein was observed, at least on western blot. Furthermore, we showed that IgT2 was readily detected in mucus and mucus-associated bacteria, but not in serum of naïve fish.

### Localization and tissue distribution of B cells in the gills, gut, spleen and head kidney

**Figure 4:**
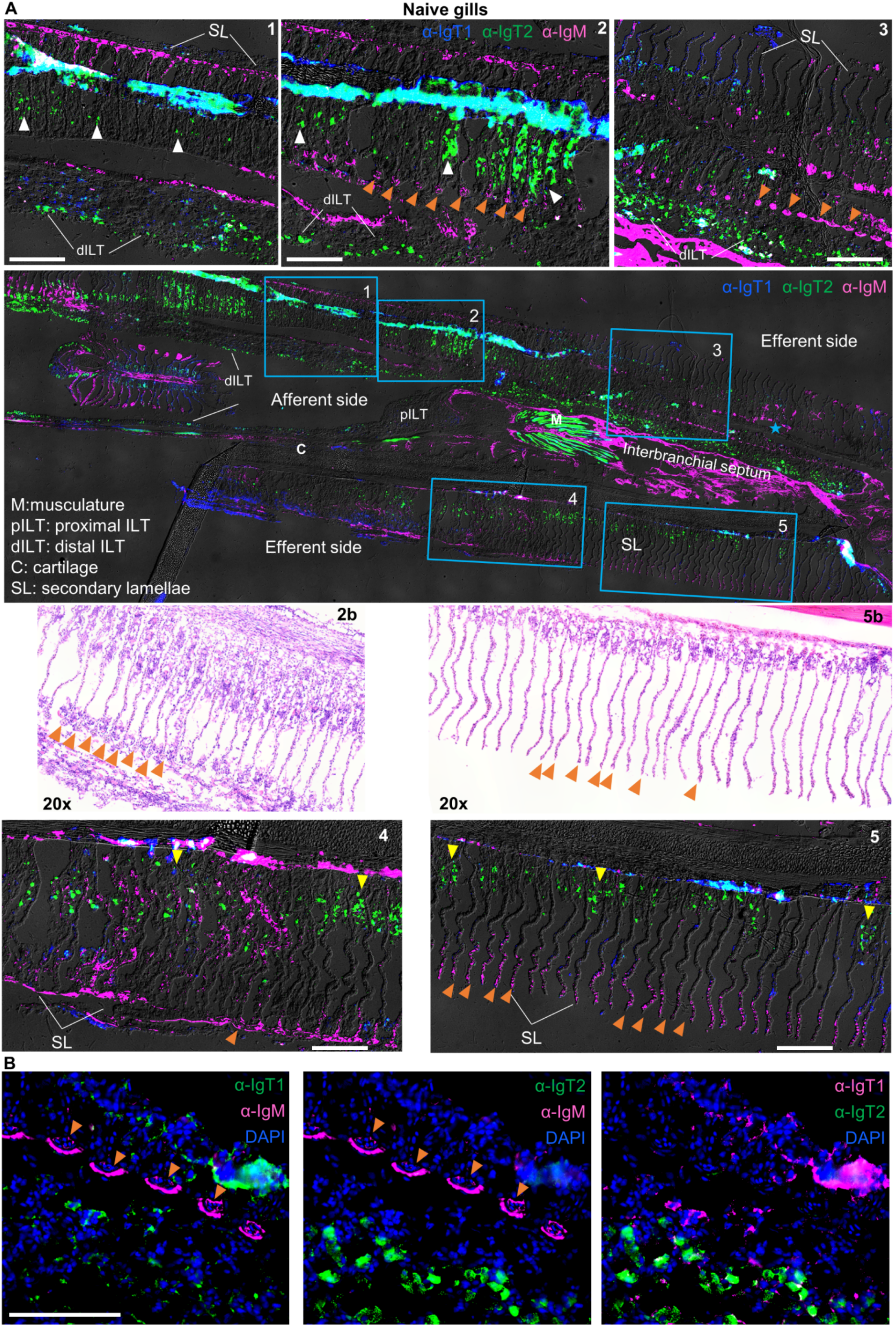
Localization and tissue distribution of B cells in the gills. **(A)** Cryosections (5 µm) of naïve gills were labelled with chicken anti-IgT1 (α-IgT1, 15 µg/ml), rabbit anti-IgT2 (α-IgT2, 5 µg/ml) and anti-IgM (α-IgM, 2 μg/ml) primary antibodies and Alexa 647-conjugated goat anti-chicken IgY (pseudo-colored blue, 4 µg/ml), TRITC-conjugated swine anti-rabbit IgG (pseudo-colored green, 1:100) and FITC-conjugated goat anti-mouse IgG (pseudo-colored magenta, 4 µg/ml). DAPI was used to stain nuclei. A tail-scan image was acquired at 20x magnification and fluorescence channels were merged with DIC to show the entire gill structure (second panel). Insets of the areas marked by blue squares are displayed (upper and lower panels). A consecutive section was stained with hematoxylin and eosin (H&E), and areas corresponding to square 2 and 5 are shown (third panel, left and right). Images were captured at 20x magnification. Note the presence of IgM^+^ B cells in the secondary lamellae, especially in capillaries (orange arrowhead). IgT1^+^ and IgT2^+^ B cells are found in the epithelium between the secondary lamellae (white arrowhead) or at the base of the secondary lamellae (yellow arrowhead) and in the distal ILT (dILT). **(B)** part of the area highlighted in square 3 was imaged at 40x magnification: (left) IgT1^+^ (green) and IgM^+^ (magenta) B cells; (middle) IgT2^+^ (green) and IgM+ (magenta) B cells; (right) IgT1^+^ B cells (magenta) and anti-IgT2^+^ B cells (green). Note, orange arrowhead indicates reactivity to IgM and IgM^+^ B cells in the capillaries of the secondary lamellae. Images are representatives of n = 3 fish. ILT: intrabranchial lymphoid tissue. Scale bars indicate 100 μm. Secondary antibody controls are shown in **Supplementary Figure 3**.

We then analysed the expression pattern of IgM and IgT in mucosal (gills and gut) and systemic (spleen and head kidney) organs by immunohistochemistry. In the gills (**Figure 4**), all three B cell types were present. In general, IgT1^+^ (blue) and IgM^+^ B cells (magenta) were observed mostly in the secondary lamellae, whereas IgT2^+^ B cells (green) were observed in the primary lamellae, secondary lamellae and the distal ILT. Specifically, IgM^+^ B cells (magenta) were almost exclusively present in the secondary lamellae especially in small capillaries (**Figures 4A, B**, some indicated by orange arrowheads), also visible in the H&E staining (insets 2b and 5b). Differently, IgT2^+^ B cells (green), were predominant and widely distributed in the gills tissue; they were present in the epithelium at the base of the secondary lamellae (some indicated by yellow arrowheads, insets 4 and 5), in the epithelium within the secondary lamellae (some indicated by white arrowheads, insets 1 and 2) and in the epithelial layer of the distal interbranchial lymphoid tissue (dILT). In contrast, IgT1^+^ B cells (blue) were less abundant and were generally found in the same compartments as IgT2^+^ B cells as well as in the secondary lamellae. Notably, no overlap was observed between IgT2^+^ and IgM^+^ B cells (**Figures 4A, B**), indicating that the cross-reactivity of the anti-IgT2 antibody to IgM observed on western blot might have been due to the presence of linear epitopes that are otherwise hidden in the native protein detected by immunofluorescence analysis, or to potential differences in glycosylation between the secreted and transmembrane IgM, or to the fact that transmembrane IgM does not express the CH4µ domain present in the secreted form, which could have been a potential target of cross-reactive antibodies. Altogether, these data revealed that IgT1^+^, IgT2^+^ and IgM^+^ B cells are all present in gill tissue, each with a distinct localization pattern, and that the newly developed antibodies are not only suitable for western blot but also immunohistochemical analysis.

**Figure 5:**
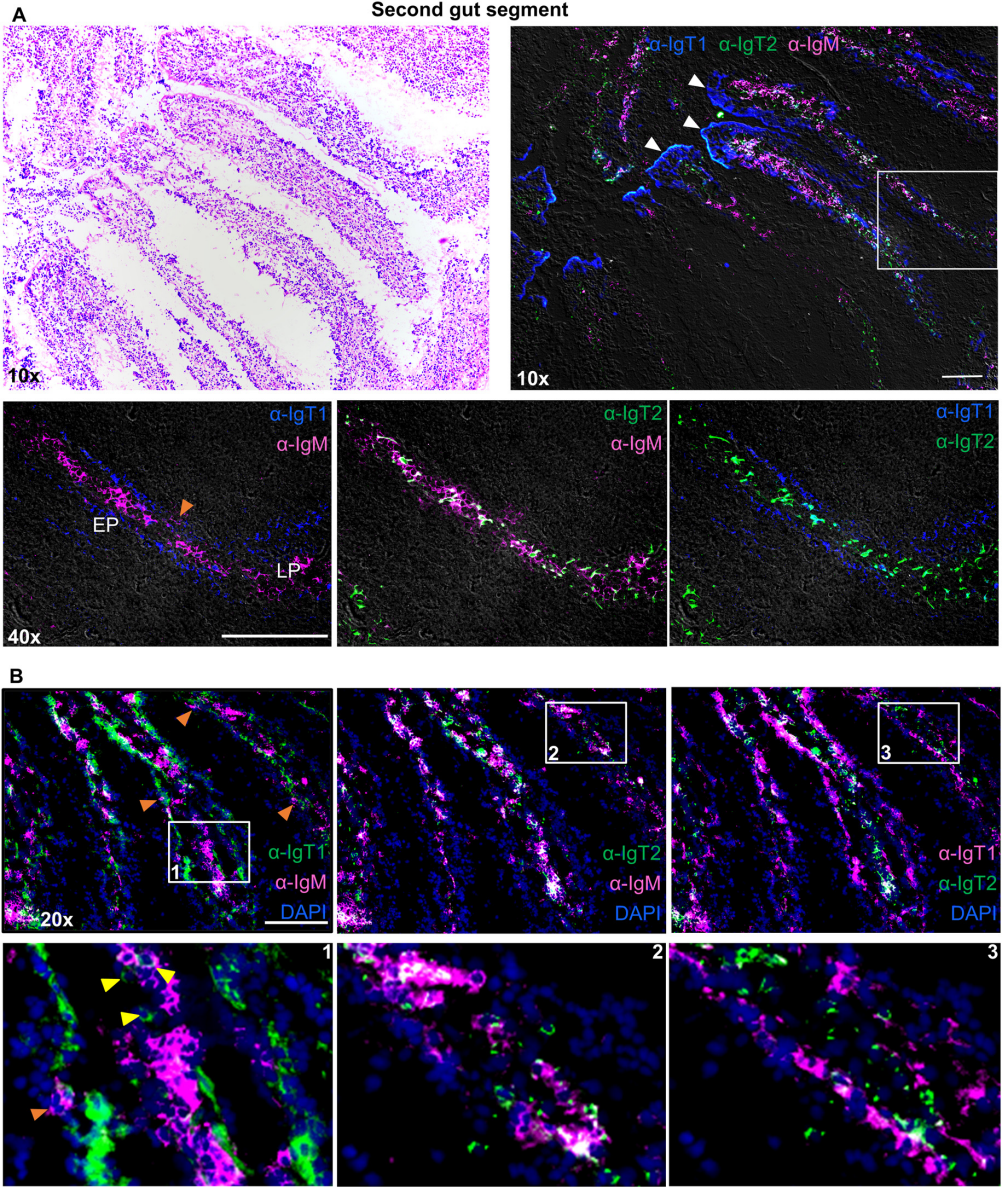
Localization and tissue distribution of B cells in the second gut segment. Cryosections (5 µm) of naïve second gut segment, were stained with hematoxylin and eosin and imaged at 10x magnifications **(A**, upper left**)**. A consecutive section of the same gut segment was labelled to visualize IgT1^+^ (blue), IgT2^+^ (green) and IgM^+^ (magenta) B cells using the same antibodies mention in **Figure 4** **(A**, upper right**)**. White arrowhead points to soluble IgT1. A selected area (white square) was imaged at 40x magnification and two-color overlays are displayed **(A**, lower panel**)**. Images were acquired using DM6b upright fluorescence microscope (Leica). LP: lamina propria; EP: epithelium. **(B)** Cryosections (5 µm) of the second gut segment were labelled with the same antibodies described in **Figure 4** and nuclei were counterstained with DAPI. Images were acquired at 20x magnifications and distribution of the indicated B cell types is displayed in each panel **(B**, upper panel**)**. Insets indicated by the white squares are displayed in the lower panel. Yellow-arrowhead point to IgT1^+^ B cells in the lamina propria and orange arrowhead point to IgM^+^ B cells in the epithelium. Secondary antibody controls are shown in **Supplementary Figure 3** and images showing the same distribution of B cells in the third gut segment are shown in **Supplementary Figure 8**. Images are representatives of n = 3 fish. Images in **(A, B)** are from two different fish. Scale bars indicate 100 μm.

Both, IgM^+^ and IgT2^+^ B cells were most abundant in the lamina propria of the second (**Figure 5**) and third (**Supplementary Figure 8**) gut segments, whereas IgT1^+^ B cells were mostly seen in the epithelium as intraepithelial lymphocytes (IELs) (**Figures 5A, B**). The tips of the villi were generally IgT1 positive (white arrowhead), possibly indicating a high concentration of soluble IgT1, in agreement with the high concentration of soluble IgT1 detected by western blot analysis of gut mucus (**Figure 2C**). To some extent, IgT1^+^ cells were also observed in the lamina propria of both gut segments or at the interface between the basal membrane of the epithelium and the lamina propria of the third gut segment (yellow arrowheads); similarly, also IgM^+^ B cells were occasionally observed in the epithelium of both gut segments (orange arrowhead) (**Figure 5**; **Supplementary Figure 3**).

**Figure 6:**
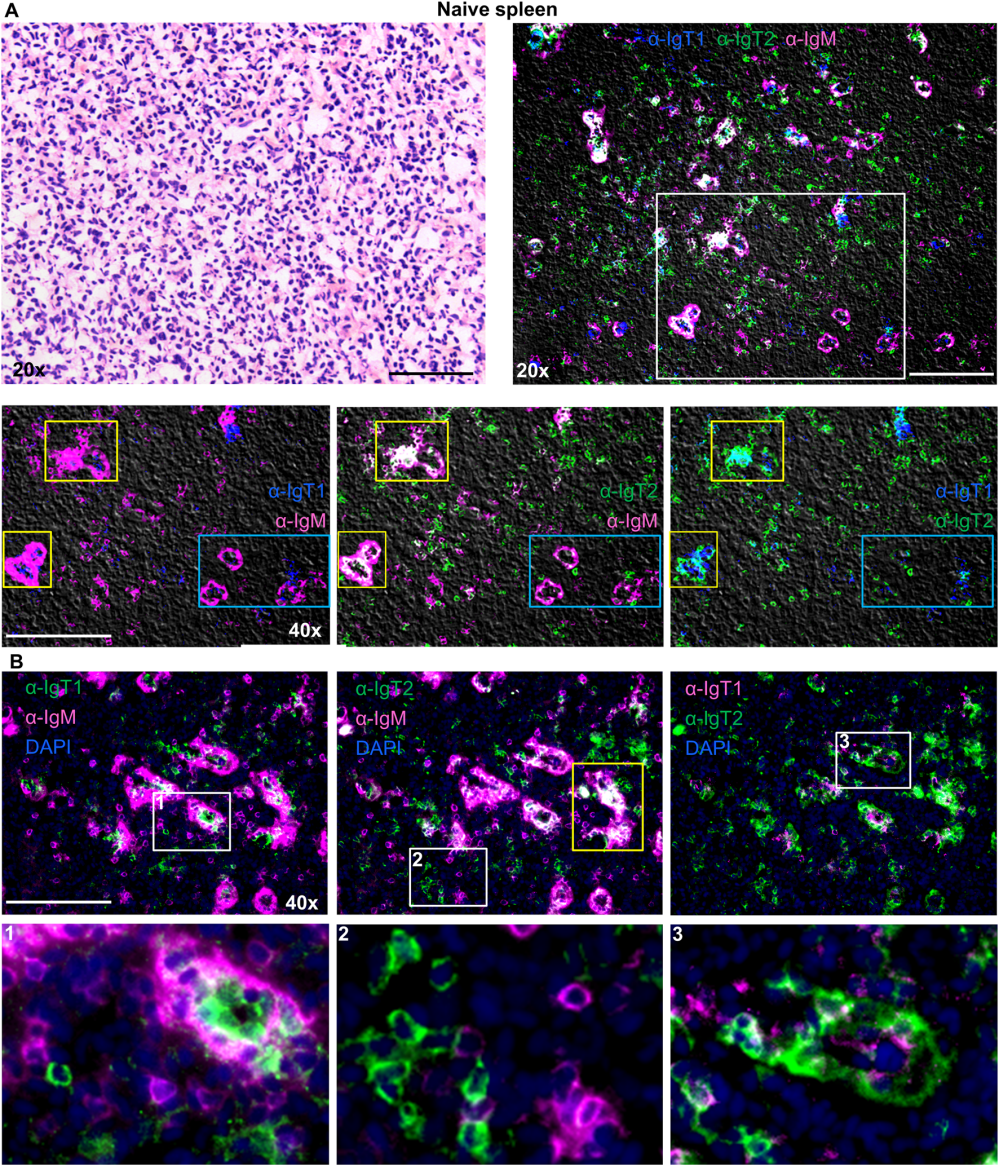
Localization and tissue distribution of B cells in the spleen. Cryosection (5 µm) of naïve spleen, was stained with hematoxylin and eosin and imaged at 20x magnifications **(A**, upper left**)**. A consecutive section was labelled to visualize IgT1^+^ (blue), IgT2^+^ (green) and IgM^+^ (magenta) B cells using the same antibodies mention in **Figure 4** **(A**, upper right**)**. A selected area (white square) was imaged at 40x magnification, and two-color overlays are displayed **(A**., lower panel**)**. Images were acquired using DM6b upright fluorescence microscope (Leica). Note the clustering of the three B cell types around the ellipsoids (yellow squares) which in some cases caused signal overlap. No signal overlap was observed in the ellipsoids when fewer cells were present (blue squares). **(B)** Cryosections (5 µm) of naïve spleen were labelled with the same antibodies described in **Figure 4** and nuclei were counterstained with DAPI. Images were acquired at 40x magnification and distribution of the indicated B cell types are displayed in each panel **(B**, upper panel**)**. Insets indicated by the white squares are displayed in the lower panel. In **B.1,** note IgT1^+^ B cells are surrounded by IgM^+^ B cells; in **B.2**, note the individual IgT2^+^ or IgM^+^ B cells; and in **B.3**, note IgT1^+^ B cells surrounded by IgT2^+^ B cells. Images are representatives of n = 3 fish. Secondary antibody controls are shown in **Supplementary Figure 4**. Scale bars indicate 100 μm.

In the spleen of naïve fish, (**Figure 6**), IgM^+^ B cells (magenta) were observed in large clusters around splenic ellipsoids and as scattered individual cells in the red pulp. IgT1^+^ cells (blue) were less abundant and present as scattered individual cells in the red pulp, as well as smaller clusters within the larger clusters of IgM^+^ cells near ellipsoids (white squares). IgT2^+^ B cells were also observed in the spleen and were mostly present as scattered single IgT2^+^ cells in the red pulp, or as ellipsoidal clusters intermingled with IgM^+^ cells. Overall IgT2^+^ cells were more abundant than IgT1^+^ cells. Co-localized IgM and IgT2 signal was observed, but only in correspondence of large ellipsoidal cluster (yellow squares). This was likely due to the presence of a high number of both, IgM^+^ and IgT2^+^ cells at these locations. If the co-localized signal was due to cross-reactivity of the anti-IgT2 antibody to IgM, this should have been apparent also on single scattered cells and in the smaller clusters with fewer IgM^+^ and IgT2^+^ cells as those indicated by the blue squares, and this was not the case (**Figures 6A, B.2**).

**Figure 7:**
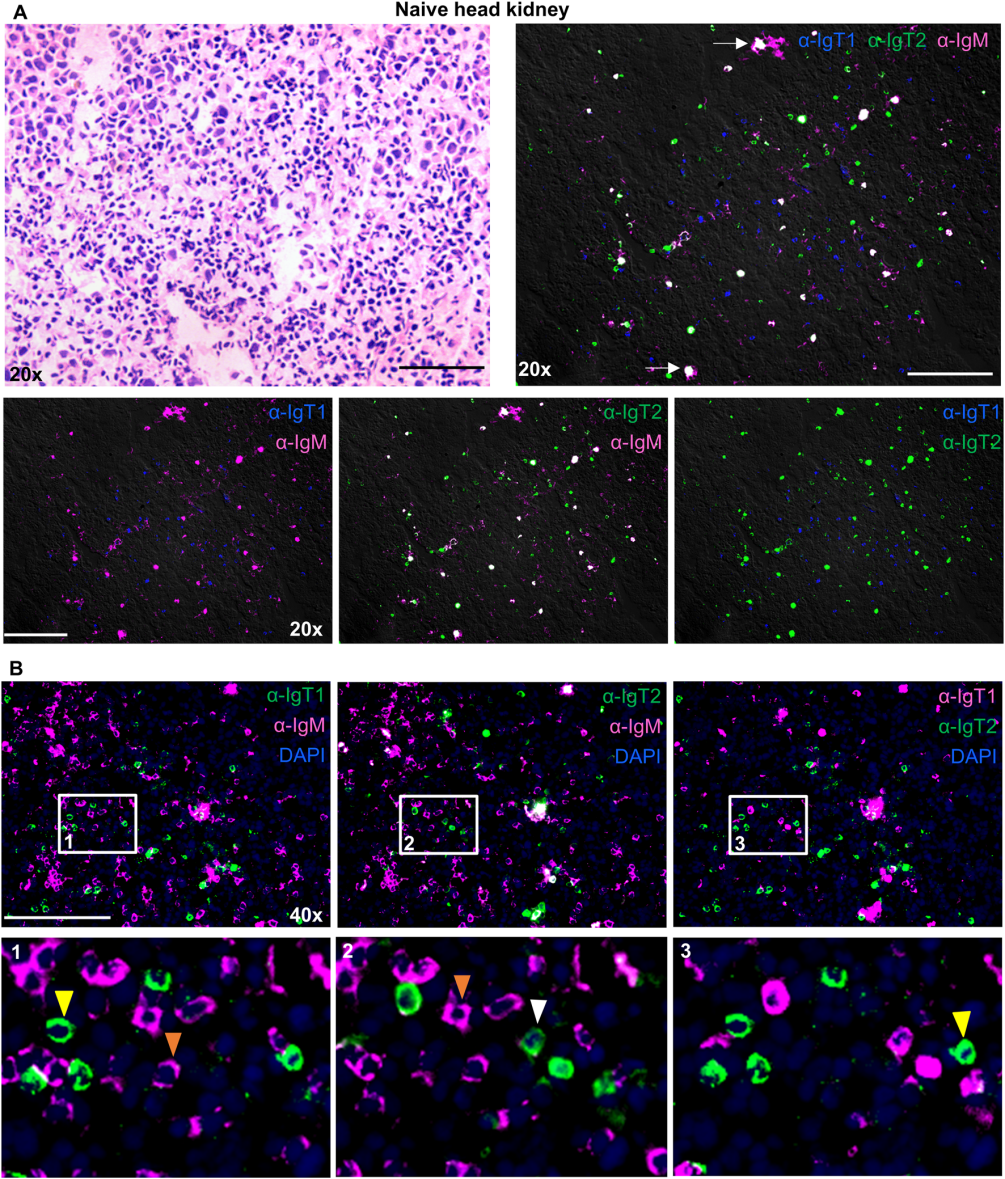
Localization and tissue distribution of B cells in the head kidney. Cryosections (5 µm) of naïve head kidney, were stained with hematoxylin and eosin and imaged at 20x magnifications **(A**, upper left**)**. A consecutive section was labelled to visualize IgT1^+^ (blue), IgT2^+^ (green) and IgM^+^ (magenta) B cells using the same antibodies mention in **Figure 4** **(A**, upper right**)**; white arrows point to areas showing IgT2 and IgM reactivity. Images were acquired at 20x or 40x magnifications using DM6b upright fluorescence microscope (Leica). **(A**, lower panel**)**, two B cell types are shown per image. **(B)** Cryosections of naïve head kidney were labelled with the same antibodies described in **Figure 4** and nuclei were counterstained with DAPI. Images were acquired at 40x magnification (Upper panel); the indicated B cell types are shown and are merged with DAPI. Insets indicated by the white squares are displayed in the lower panel. Yellow or white arrowheads point to single IgT1^+^ or IgT2^+^ B cells, and orange arrowhead point to single IgM^+^ B cells. Images are representatives of n = 3 fish. Secondary antibody controls are shown in **Supplementary Figure 4**. Scale bars indicate 100 μm.

In the head kidney (**Figure 7**), all B cell isotypes were present and were randomly scattered throughout the tissue with few small clusters of IgM^+^ B cells, but no specific organization of B cells was observed. Even though most cells were present as single positive B cells, bright spots double positive for IgT2 and IgM were observed. We hypothesized that these could either be plasma cells producing high levels of secreted IgM (containing the CH4µ domain), B cells in rearrangement expressing peptides for both, IgT2 and IgM, or melanomacrophages (MM) having trapped a large amount of IgT2 and IgM. The latter however is unlikely because no overlap with IgT1 was observed, and there is no reason why MM would not trap IgT1 as well. Altogether, by using immunofluorescence, all three types of B cells were detected in mucosal as well as systemic organs of common carp with various distribution patters.

### B cell responses during infection with the blood-borne parasite *Trypanoplasma borreli*

We next investigated whether IgT1^+^ and IgT2^+^ cells in secondary lymphoid organs would react to a systemic infection as the one triggered by the extracellular eukaryotic parasite *T. borreli*, which can infect common carp and divides in its blood and tissue fluids. To this end, spleen (a typically heavily affected organ (44)) collected from *T. borreli*-infected carp at the peak of parasitaemia (3 weeks post-infection) was examined by immunofluorescence, and serum collected at the same time point after infection was examined for the presence of parasite-specific immunoglobulins by western blot.

**Figure 8:**
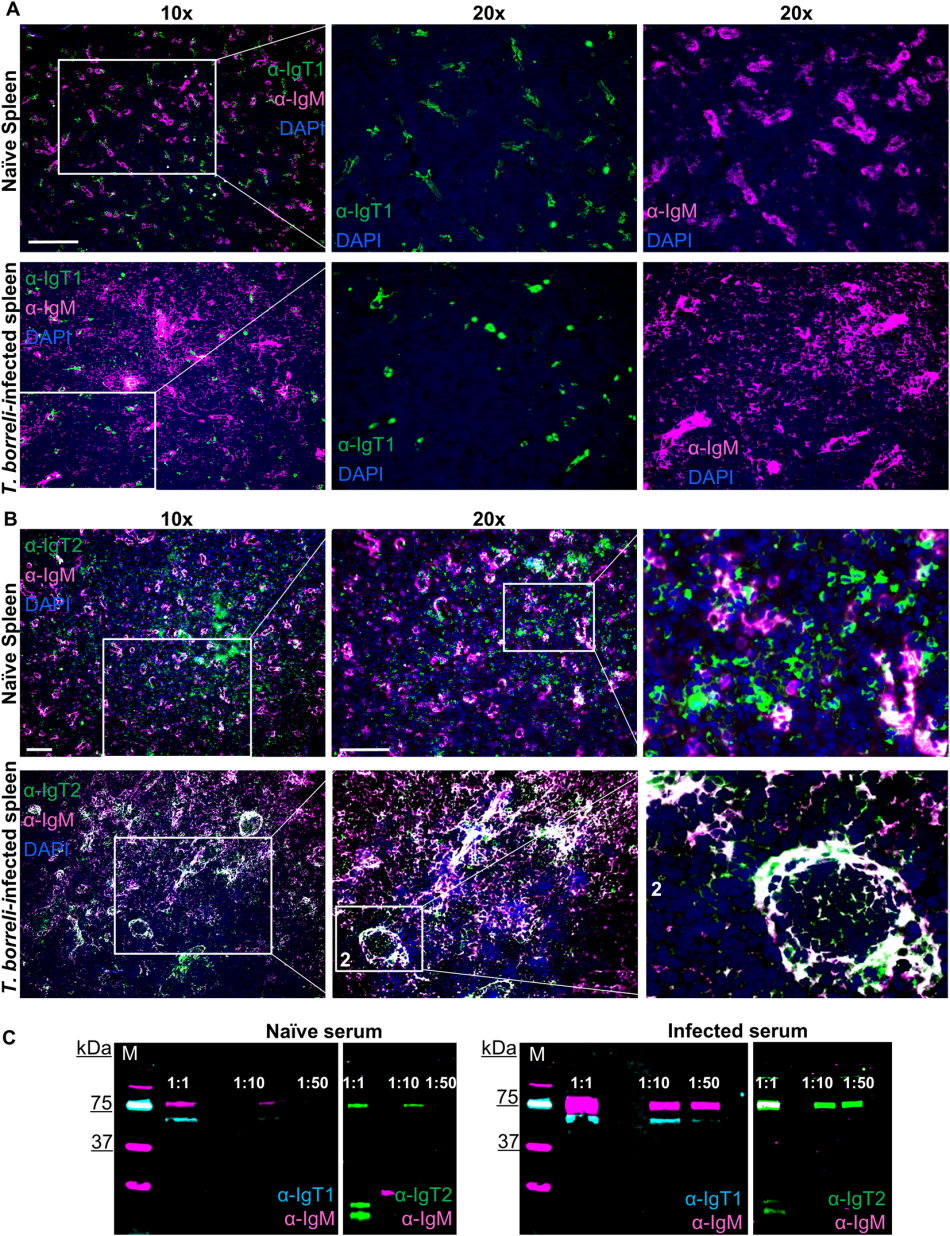
B cell responses during infection with the blood-borne parasite *T. borreli.* Spleen was isolated from naïve carp or from 3-weeks infected carp. Cryosections (5 µm) were labelled for anti-IgT1 and anti-IgM **(A)** or anti-IgT2 and anti-IgM **(B)**, as described in **Figure 4** and nuclei were counterstained with DAPI. Images were acquired using DM6b upright fluorescence microscope (Leica). Images are representatives of n = 3 fish. Scale bars indicate 100 μm. Note the expansion of IgM^+^ B cells in the infected spleen **(A, B**, lower panels**)**. No apparent difference in the number IgT1^+^ and IgT2^+^ B cells in the infected spleen when compared to the non-infected spleen was observed. **(C)** Western blot analysis of *T. borreli*-specific antibodies. Parasites were incubated for 1h with 1:1, 1:10 and 1:50 dilutions of serum from naïve (left) or 3-weeks infected carp (right). Parasites were washed, fixed and boiled for western blot analysis using chicken anti-IgT1 (α-IgT1: 10 µg/ml) or rabbit anti-IgT2 (α-IgT2, 2.5 µg/ml), both in combination with mouse-anti-IgM (α-IgM, 2 μg/ml), followed by IRDye800-conjugated donkey anti-chicken IgY (pseudo-colored cyan) or IRDye800-conjugated goat anti-rabbit IgG (pseudo-colored green) and Alexa 647-conjugated goat anti-mouse-IgG (pseudo-colored magenta) secondary antibodies. Note the higher IgT1 and IgM signal when parasites were incubated with infected serum (right) compared to the parasite incubated with naïve serum (left) and the absence of IgT2 signal in either naïve (left) or *T. borreli*-infected serum (right). Images were captured with the infrared florescence imager (Odyssey).

In agreement with previous reports (44), immunofluorescence analysis showed a clear expansion of IgM^+^ B cells in spleen from infected compared to non-infected fish (**Figure 8**). In contrast, the number and distribution of IgT1^+^ B cells did not seem to significantly differ between infected and non-infected tissue (**Figure 8A**). In non-infected spleen, as observed earlier, IgT2^+^ B cells were numerous, clustered together with IgM^+^ B cells in correspondence of ellipsoids and were also present as scattered single cells in the red pulp (**Figure 8B**, upper panel). In infected tissue however, we observed a significant overlap between the IgM and IgT2 signal (**Figure 8B**, lower panel). Based on our observations in other organs and on the western blot analysis, this is likely due to the significant increase in the production of secreted IgM and the possibility of cross-reactivity of our anti-IgT2 to soluble IgM. When focusing on the single positive IgT2 cells (green), no obvious increase in IgT2^+^ B cells was observed. To be able to assess whether the changes observed in the spleen were reflected in changes in circulating Ig, we examined serum from infected fish for the presence of *T. borreli*-specific immunoglobulins. To this end, parasites were incubated with different dilutions of serum from non-infected (naïve serum) or *T. borreli*-infected carp, followed by western blot analysis. In naïve serum, low amounts of pre-existing IgM and IgT1 and no IgT2 specific for the parasites were observed (**Figure 8C**, left panels). In serum from infected fish, an increase in parasite-specific IgM and IgT1 was observed, as IgT1 and IgM signal was visible up to 1:50 serum-dilution, and again, no parasite-specific IgT2 was detected (**Figure 8C**, right panels). These results show the presence of *T. borreli*-specific IgT1 and IgM and the absence of IgT2 specific to the parasite, indicating that IgT1 and not IgT2 may be potentially involved in systemic immune responses in carp.

## Discussion

In this study, polyclonal antibodies against carp IgT1 and IgT2 were developed in chicken and rabbit, respectively. The specificity of the two polyclonal antibodies was investigated and confirmed by various methods, including peptide blocking and MS analysis. The location of B cells expressing IgM, IgT1 and IgT2 was then investigated, as well as their serum titers. Our results revealed the presence of soluble IgT1 and soluble IgM in both, systemic and mucosal compartments, while the expression of soluble IgT2 is restricted to mucosal tissues.

Two IgT subtypes named IgZ1 and IgZ2 were initially described in (East-Asian) common carp (9), based on predictions from genomic sequences. Both blast analysis of the current carp genome and RT-PCR from European common carp led to the confirmation of the IgZ2 sequence with 2 constant domains (here named IgT2). In contrast, we could not find the full IgZ1 sequence in the genome and we failed to amplify it from our samples. Instead, we found in the same contig four exons encoding a IgT heavy chain with four constant domains, which we named IgT1; this sequence was highly similar (about 90% similarity of AA sequences) to the recently reported IgZ3 (46). The expression of this sequence was validated by RT-PCR from several tissues. For the time being, we therefore conclude that European common carp expresses two IgT subtypes with 4 and 2 constant domains respectively. The CH1 domain sequences of IgT1 and IgZ3 are highly similar to IgT2 CH1 as well as to IgM CH1. The IgT2 CH2 domain is most similar to IgT1, IgZ3 and IgM CH4 domains, as previously reported (9, 15, 46). These conclusions based on genome sequence and mRNA expression were further validated using two polyclonal antibodies directed against IgT1 and against IgT2. The reactivity of these antisera was first analysed in western blot. Both anti IgT1 and anti IgT2 recognized protein with expected theoretical molecular weights of 61 kDa (for IgT1), and 50kDA (for IgT2) from serum and mucus samples. The anti IgT1 antiserum also recognized proteins of other molecular weight (around 79 kDa, 50 kDa, and 35 kDa). These peptides could be either degradation products of IgT1 by proteases, or splicing variants. In fact, two IgT1-related sequence variants were reported in the full-body transcriptome of carp (17). One variant was composed of three CH domains (CH1τ_1_, CH3τ_1,_ and CH4τ_1_) lacking the CH2τ_1_ domain (KTF75694). Nevertheless, the fact that the three CH domains of the shorter variant are identical to the corresponding domains of the complete IgT1, makes it more likely a product of alternative splicing rather than a product of an IgT1 gene lacking the exon coding the CH2 domain. Furthermore, during our genome search, we found no evidence of scaffolds containing tree instead of four IgT1-related CH domains. The other sequence predicted from the whole-body transcriptome was composed of six CH domains (KTG39838), with a CH1-like- CH4-like- CH3-like- CH4-like- CH3-like-CH4-like structure which does not fit the known IgT loci of the carp genome. Since we always failed to amplify the transcript of this isoform, we cannot explain the reactivity of the anti-IgT1 to a peptide of ~79 kDa by the recognition of such 6xCH IgT heavy chain. If expressed, the two IgT1-related proteins could all likely be recognized by our anti-IgT1 antibody due to the ubiquitous presence of a highly conserved CH3 domain.

Using anti-IgM, -IgT1 and -IgT2 Abs, soluble IgT1 and IgM were found in the serum, whereas IgT2 was not detected. While the anti-IgT1 polyclonal antiserum was very specific for this subtype, anti-IgT2 showed cross-reactivity to IgM. This cross reactivity to IgM was possibly due to the high similarity between CH1τ_2_ and CH1μ of IgM, or between CH2τ_2_ and CH4µ. However, as the first constant domain of IgT1 is also highly similar to CH1µ, the lack of cross-reactivity of the anti-IgT1 poly-serum with IgM is in favor of the second hypothesis. Furthermore, high levels of co-staining of B cells with anti-IgM and anti-IgT2 appears to be restricted to conditions in which secreted Abs are present, as in *T. borreli*-infected tissues. In fish, the fourth constant domain is typically removed from the membrane bound form of the μ heavy chain by splicing to TM exons and is therefore only present in secreted IgM. In fact, our anti-IgM antibody recognized a 50 kDa protein corresponding to a shorter IgM molecule, when IgM+ B cells sorted from carp peripheral blood leukocytes are analysed by western blot (data not shown). Overall, we conclude that several observations suggest that the anti-IgT2 cross reactivity is likely directed against the CH4 domain of secreted IgM.

Overall, our data point to a role for soluble IgT1 and IgM in both systemic and mucosal compartments, and to a role for soluble IgT2 exclusively at mucosal surfaces; IgM, IgT1 and IgT2 were detected by WB in mucus samples. Our results in carp are in accordance with recent studies conducted in zebrafish, where IgZ1 was observed in serum, skin and gills mucus, but IgZ2 only in skin and gill mucus (32). The expression pattern of IgT1 was most similar to the one described for trout IgT, with detectable levels in serum, and highest levels in gut mucus (25), compared to skin (26) or gills mucus (29). Carp IgT1 appears to be the main Ig in the gut, with higher levels than IgM and IgT2. We also observed that all three carp Ig types were involved in coating of microbiota isolated from skin, gills, and gut mucus, as previously reported in zebrafish and rainbow trout (32, 47).

We also used the anti IgT1 and IgT2 Abs to study the distribution of B cell subtypes using immunofluorescence. In naïve spleen, B cells were found either as single scattered cells throughout the red pulp, or organized in clusters around the numerous ellipsoids. Most frequently, clusters of IgT1^+^ cells were surrounded by clusters of IgM^+^ and IgT2^+^ B cells. The anti-IgT2 antibody showed a distinct pattern of cross-reactivity to IgM^+^ areas and it clearly did not cross-react to all IgM^+^ B cells. Additionally, cross-reactivity was restricted to the luminal side of ellipsoids (where IgM secretion to the blood may occur), or to discrete scattered large spots within the head kidney (where plasma cells or maturing B cells may reside), again supporting the idea that anti IgT2 cross reactivity to IgM likely targets sIgM CH4. Ellipsoids are small splenic capillaries in which, in carp, not only B cells but also T cells (personal observation) and other leukocytes, including melanomacrophages, can be found. These areas are antigen-trapping zones, and may corresponds to recently reported MLA, which are functional counterparts of germinal centers in fish (48). The colocalization and clustering of carp splenic B cells shows a clear form of organization of white blood cells in the carp spleen.

In the gut, we observed a restricted localization of IgT2^+^ and IgM^+^ B cells in the lamina propria (LP), with few IgM^+^ B cells found as intra-epithelial lymphocytes (IELs). In contrast, IgT1^+^ B cells were primarily found as IELs, and only a few were found in the LP especially in the third gut segment. An early study in carp already showed the presence of IgM^+^ B cells in the LP and presence of IgM-negative but further undefined IELs in the intestine of carp (49). Later studies conducted in trout orally vaccinated against or infected with the gut parasite *Ceratomyxa shasta*, described the presence of IgM^+^ and IgT^+^ B cells in the lamina propria, and described IgT^+^ B cells in the epithelium as IELs, also secreting soluble IgT (50). Our findings in carp appear in agreement with the latter study. The exclusive presence in carp of IgT1^+^ B cells in the epithelium, and the high amount of soluble IgT1 observed in the gut lumen detected by immunofluorescence, or in gut mucus or mucus-associated bacteria detected by western blot, all points towards a role for IgT1 in intestinal, local immune responses and immune tolerance.

In the gills, both IgT1^+^ and IgT2^+^ B cells were widely distributed, and IgT2^+^ appeared to be the dominant B cell type overall in contrast to the intestine. The dominance and wide distribution of IgT2^+^ B cells especially at the epithelial surfaces of the gills, likely hints at an important role of IgT2^+^ B cells in local immunity. As soluble IgT2 is present only in the gills in non-glycosylated form, it will be interesting to investigate the functional difference between the non-glycosylated IgT2 from the gills and the glycosylated IgT2 from other mucosal compartments (skin and gut). Whether IgT2^+^ B cells may play a greater role in gills-specific immune responses and IgT1^+^ B cells are most involved in gut-related immune responses, awaits further investigation.

In serum of carp infected with a blood parasite, western blot analysis revealed the presence of *T. borreli*-specific IgT1 and IgM antibodies in the serum, while IgT2 was not detectable. This confirmed earlier observations (9), pointing at a role for both, IgT1 and IgM in systemic immune responses against the same *T. borreli* blood parasite, and at a role for IgT2 in responses to mucosal infection with the *Lernea* anchor worm. These findings in carp are in line with results obtained in zebrafish in response to *Edwardsiella tarda* showing that IgZ1 levels increased in serum at 7 days post infection, while IgZ2 was totally absent (32). Nevertheless, IgM^+^ B cells remain the prominent B cell type responding to systemic infection and considering the abundance of IgT2+ B cells in the spleen, it cannot be completely excluded that also IgT2+ B cells can take part in systemic responses, perhaps with other pathogens than those investigated thus far. A role of IgT in response to systemic infection is also consistent with observations in rainbow trout and other teleost species (20, 32–35).

In conclusion, in this study we characterized two newly developed polyclonal antibodies against heavy chain of the IgT subtypes in common carp. We describe a differential expression pattern of soluble immunoglobulins in systemic (IgM and IgT1) and mucosal compartments (IgM, IgT1 and IgT2). Compartmentalization of B cells was observed in gills and gut with specific Ig isotype/subtype expression, and splenic B cells were shown to organize themselves around ellipsoids. Our antibodies pave the way for future studies aiming at the characterization of humoral immune responses of carp to infections and vaccinations. For example, follow up *in vivo* studies using gills and gut infection models will help elucidating the function of each immunoglobulin isotype in response to different mucosal infections and will further improve our understanding of mucosal humoral immunity in carp.

## Data Availability

The original contributions presented in the study are publicly available. This data can be found here: GenBank, accession number PQ441757.
