# Supplementary material for: Distinct distribution and responses of IgM+, IgT1+ and IgT2+ B cells in common carp
Source: Front Immunol. 2024 Nov 11;15:1490776. doi: 10.3389/fimmu.2024.1490776 (PMC11586371; doi:10.3389/fimmu.2024.1490776)
Supplement: Supplementary file 1 [file DataSheet1.pdf]

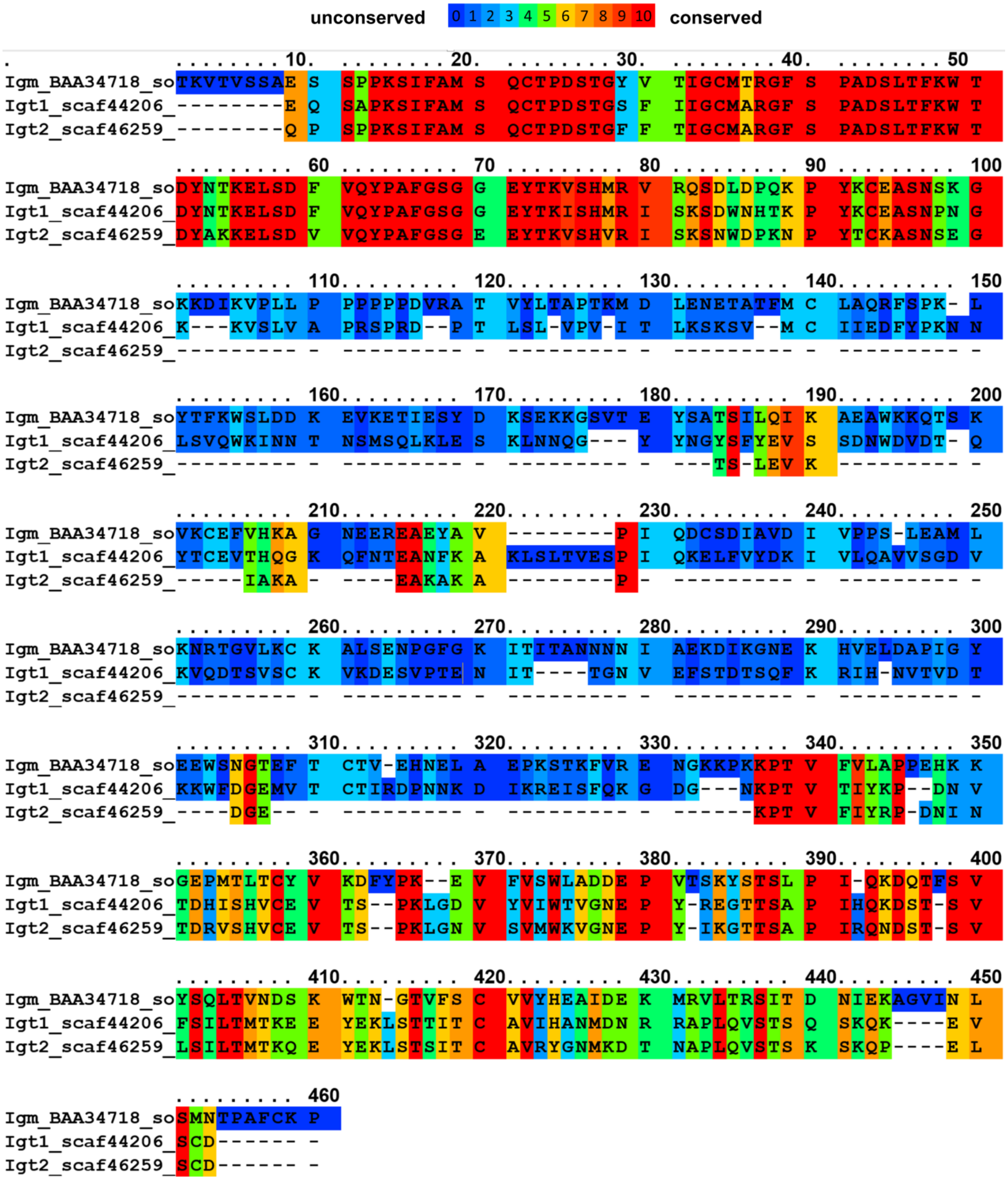

Supplementary Figure 1. Protein sequence alignment between European carp IgT1, IgT2 and secreted IgM (BAA34718). Multiple sequence alignment was performed using PRALINE with the default parameters. Note the high degree of similarity among the CH1 domain of all sequences.

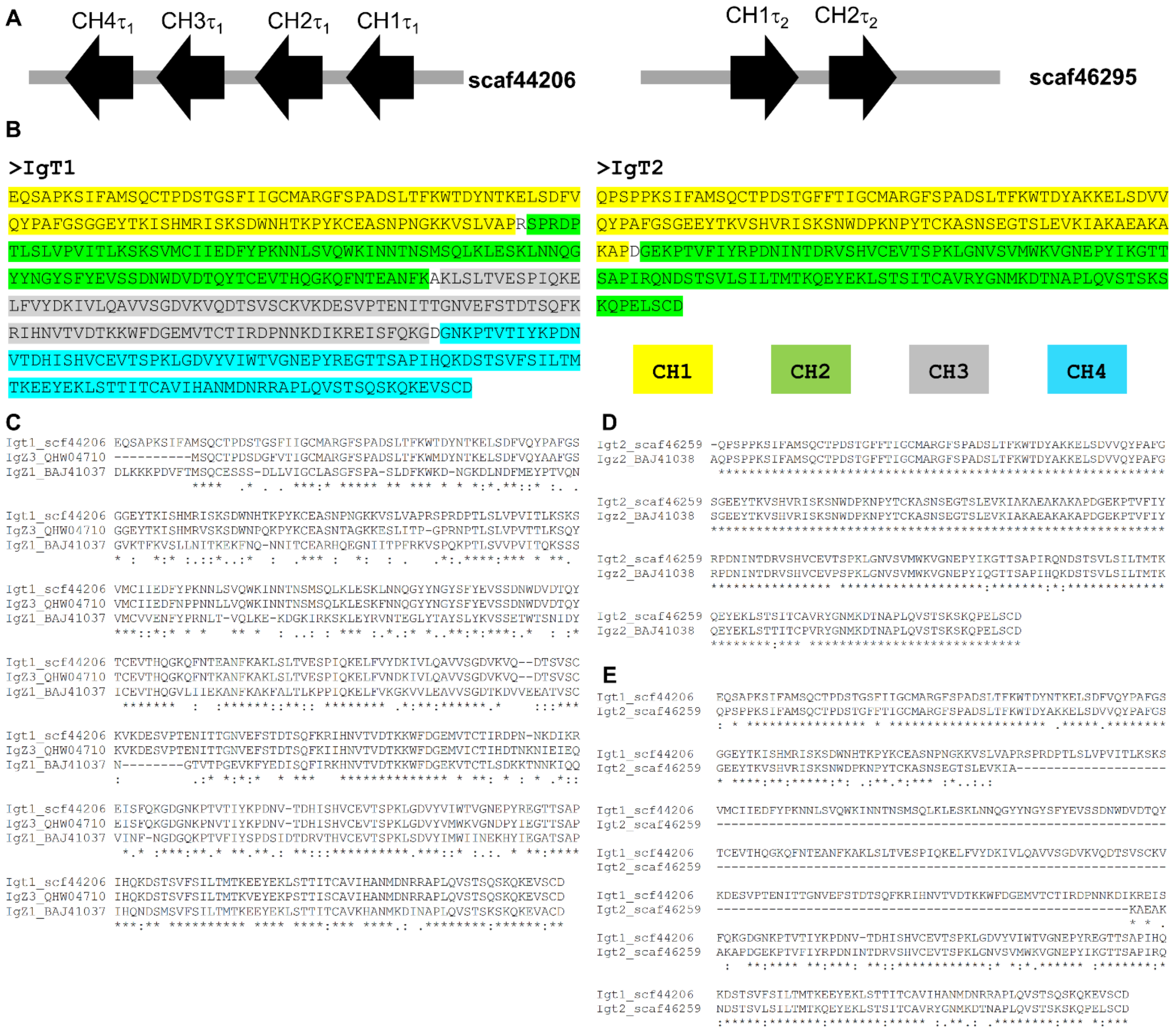

Supplementary Figure 2. (**A**) Schematic representation of the position of the exons retrieved on two scaffolds of the European carp genome showing highest similarity to the sequences of the East-Asian carp IgZ1 (AB598367) and IgZ2 (AB598368). (**B**) Protein sequence of European carp IgT1 and IgT2 with the constant immunoglobulin domains (CH) indicated by the indicated colours. (**C**) protein sequence alignment between IgT1 sequence retrieved on scaffold44206 and the published East-Asian carp IgZ1 (BAJ41037) and IgZ3 (QHW04710) sequences. (**D**) protein sequence alignment between IgT2 sequence retrieved on scaffold44295 and the published East-Asian carp IgZ2 sequence (BAJ41038). (**E**) protein sequence alignment between European carp IgT1 and IgT2. All multiple sequence alignments were performed with Clustal omega with the default parameters.

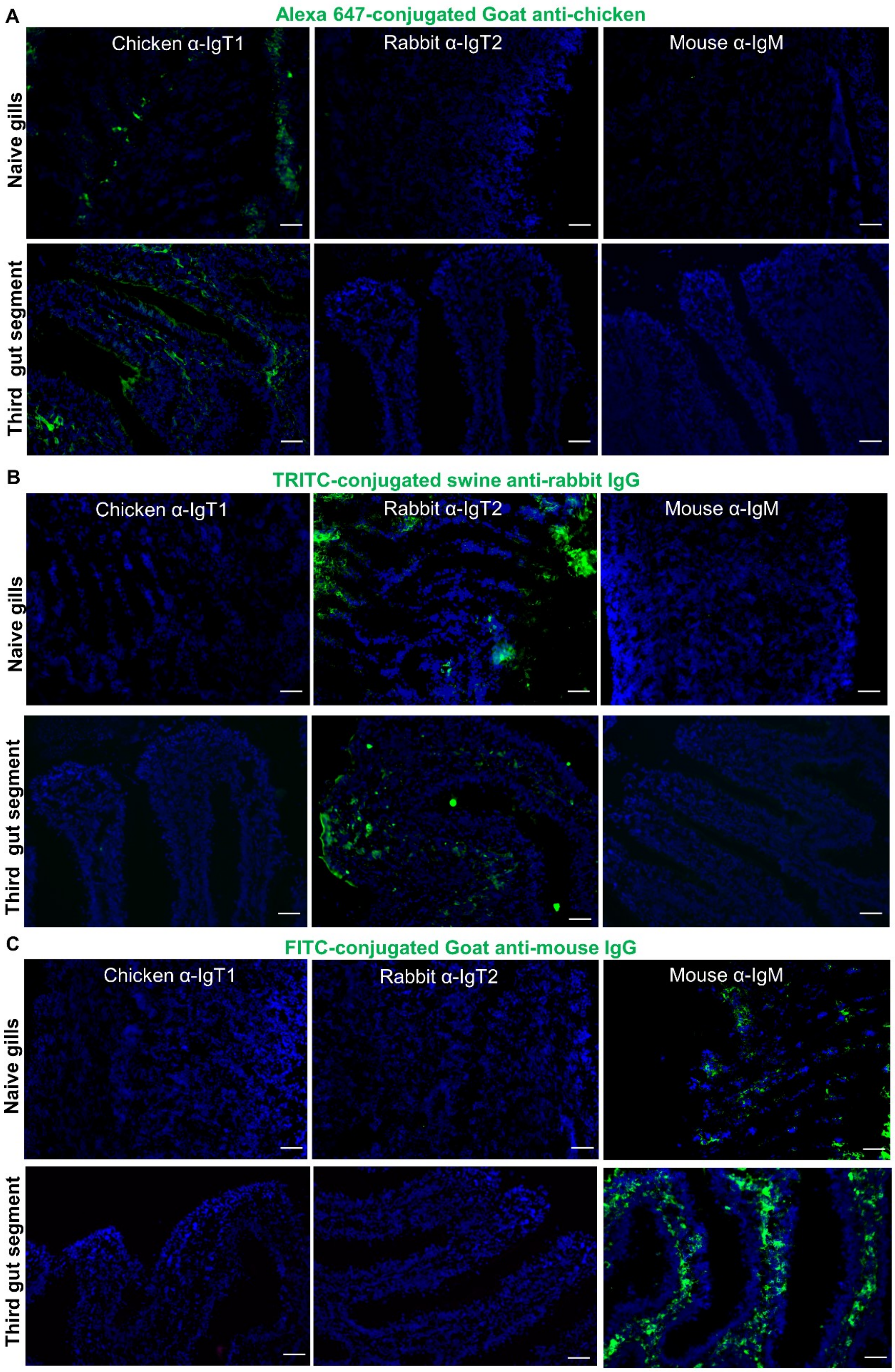

Supplementary Figure 3. Specificity of the secondary antibodies used for immunofluorescence analysis. Cryosections (5 µm) of gills and gut (third segment) were incubated with the indicated primary antibody (chicken anti-IgT1 (15 µg/ml) or rabbit anti-IgT2 (5 µg/ml) or mouse anti-IgM (1:100), followed by incubation with a single secondary antibody specific for only one of the three primary antibodies. (**A**) Incubation with Alexa 647-conjugated goat anti-chicken IgY, (4 µg/ml) (**B**), Incubation with TRITC-conjugated swine anti-rabbit IgG (1:100) (**C**), Incubation with FITC-conjugated goat anti-mouse IgG, (4 µg/ml). Nuclei were stained with DAPI (blue). Note, a specific signal is present only when secondary antibodies were incubated with the relevant primary antibody. Images were acquired at 20x magnification using DM6b upright fluorescence microscope (Leica). Scalebar indicates 50 µm.

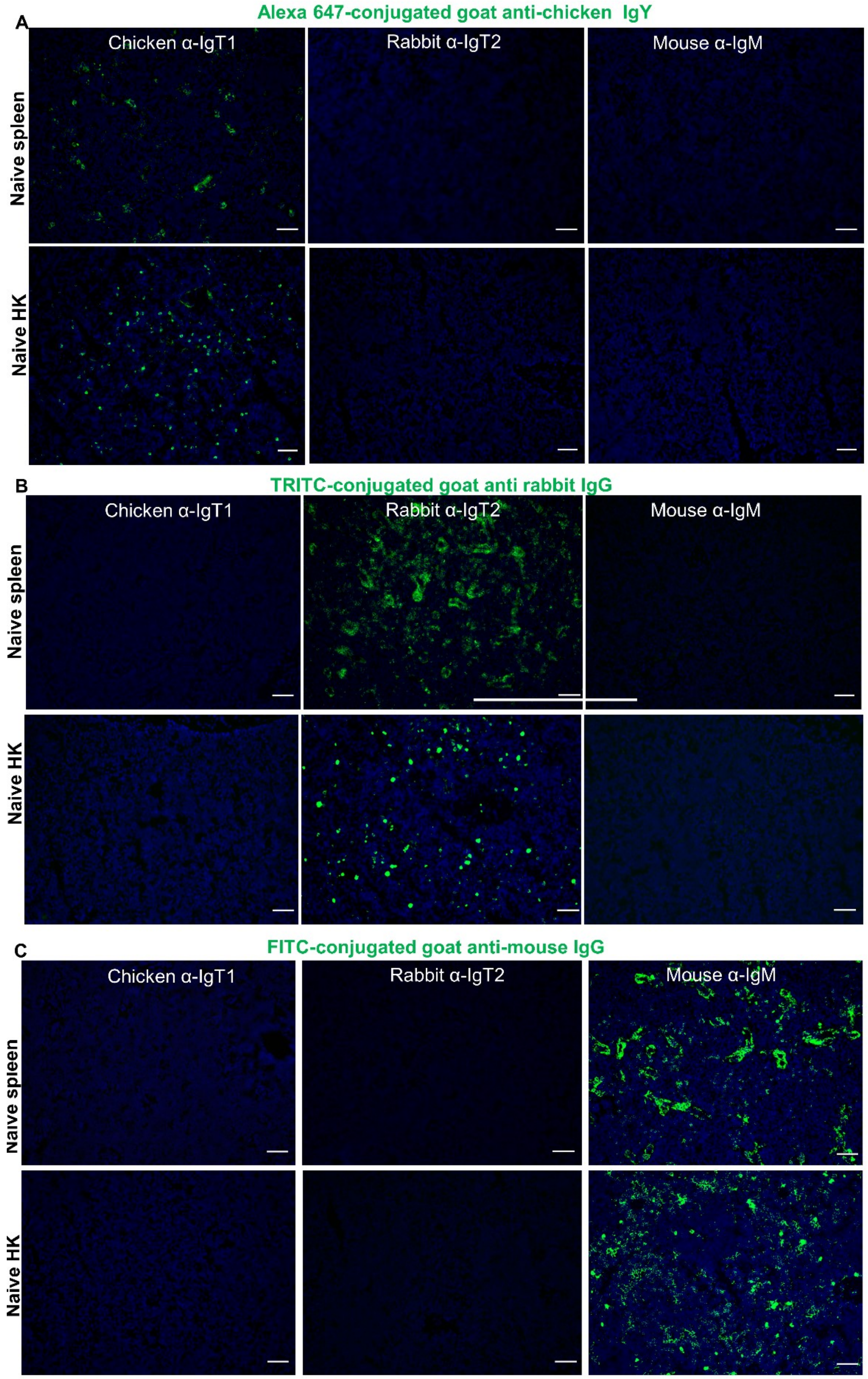

Supplementary Figure 4. Specificity of the secondary antibodies used for immunofluorescence analysis. Cryosections (5 µm) of head kidney and spleen were incubated with the indicated primary antibody (chicken anti-IgT1 (15 µg/ml) or rabbit anti IgT2 (5 µg/ml) or mouse anti-IgM (1:100), followed by incubation with a single secondary antibody specific for only one of the three primary antibodies. (**A**) Incubation with Alexa 647-conjugated goat anti-chicken IgY, (4 µg/ml) (**B**), Incubation with TRITC-conjugated swine anti-rabbit IgG (1:100) (**C**), Incubation with FITC-conjugated goat anti-mouse IgG, (4 µg/ml). Nuclei were stained with DAPI (blue). Note, a specific signal is present only when secondary antibodies were incubated with the relevant primary antibody. Images were acquired at 20x magnification using DM6b upright fluorescence microscope (Leica). Scalebar indicates 50 µm.

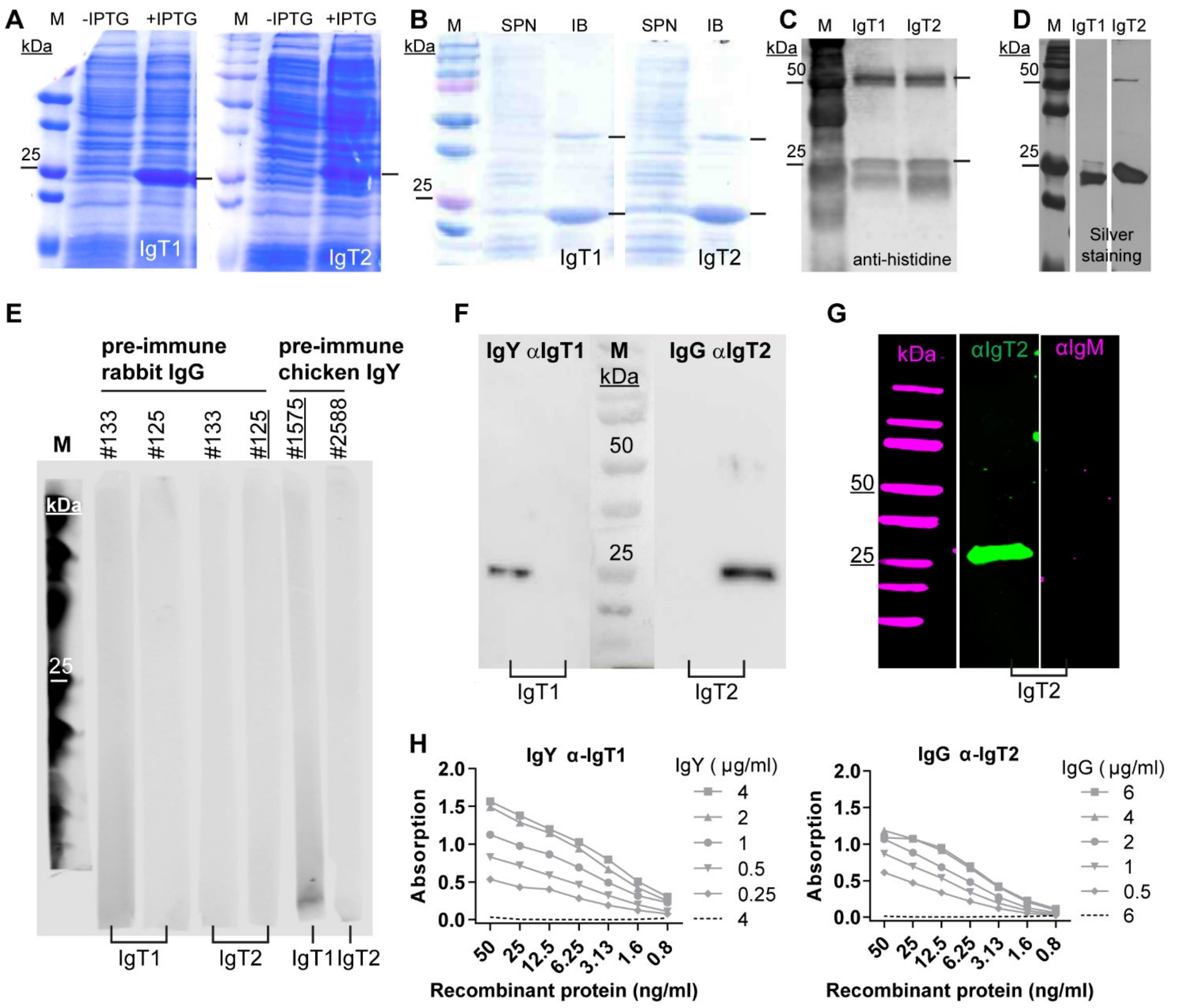

Supplementary Figure 5. Recombinant IgT1 and IgT2 production, purification and validation of antibody specificity. (**A-B**) IgT1 and IgT2 were produced in bacteria after 4h induction with IPTG. Soluble bacterial proteins in lysate supernatant (SPN) were separated from inclusion bodies (IB). Recombinant IgT1 and IgT2 were purified from the solubilized IB under denaturing conditions and purified by Ni^{2+}-NTA affinity chromatography. (**C-D**) Protein specificity was assessed by western blot analysis on a 4-20% SDS-PAGE under reducing conditions, using the anti-penta-histidine mouse monoclonal antibody (1:5000) as primary antibody and HRP-conjugated Goat anti-mouse (1 µg/ml) as secondary antibody. In parallel, purity of the eluted, refolded proteins (200 ng/lane) was assessed by silver staining. Protein of the expected molecular weight of 25 kDa and 50 kDa, corresponding to the monomeric and dimeric form, were observed. (**E**) Total IgG from two pre-immune rabbits, and total IgY from two pre-immune chicken eggs, were screened by western blot analysis for absence of cross-reactivity to the indicated recombinant proteins. Rabbit #125 and chicken #1575 (underlined) were selected for immunization. (**F-G**) The indicated recombinant proteins (100 ng/lane) were resolved on a 4-20% SDS-PAGE under reducing conditions and detected using chicken anti-IgT1 (IgY αIgT1, 10 µg/ml), rabbit anti-IgT2 (IgG αIgT2, 2.5 µg/ml) (**F**) or rabbit anti-IgT2 (αIgT2) and mouse anti-IgM (αIgM, 2 µg/ml) (**G**) as primary antibodies, and HRP-conjugated rabbit anti-chicken (1:4000) or HRP-conjugated goat-anti-rabbit (1:2000) (**F**) or IRDye800-conjugated donkey anti-chicken (0.1 µg/ml, pseudo-coloured cyan), IRDye800-conjugated goat anti-rabbit (0.1 µg/ml, pseudo-coloured green) or alexa-680-conjugated goat anti-mouse-IgG (0.1 µg/ml, pseudo-coloured magenta) (**G**) as secondary antibodies. Western blot analysis confirms specificity of anti-IgT1 to recombinant IgT1, and of anti-IgT2 to recombinant IgT2 as well as absence of cross-reactivity of anti-IgM to IgT2. Images were captured with infrared florescence imager (Odyssey). (**H**) ELISA Plates were coated with the indicated concentrations of recombinant proteins. Various concentrations of anti-IgT1 (left) or anti-IgT2 (right) antibodies were used to detect the autologous recombinant proteins (full lines), or the heterologous recombinant protein (dotted lines). Absorption was measured at OD405nm.

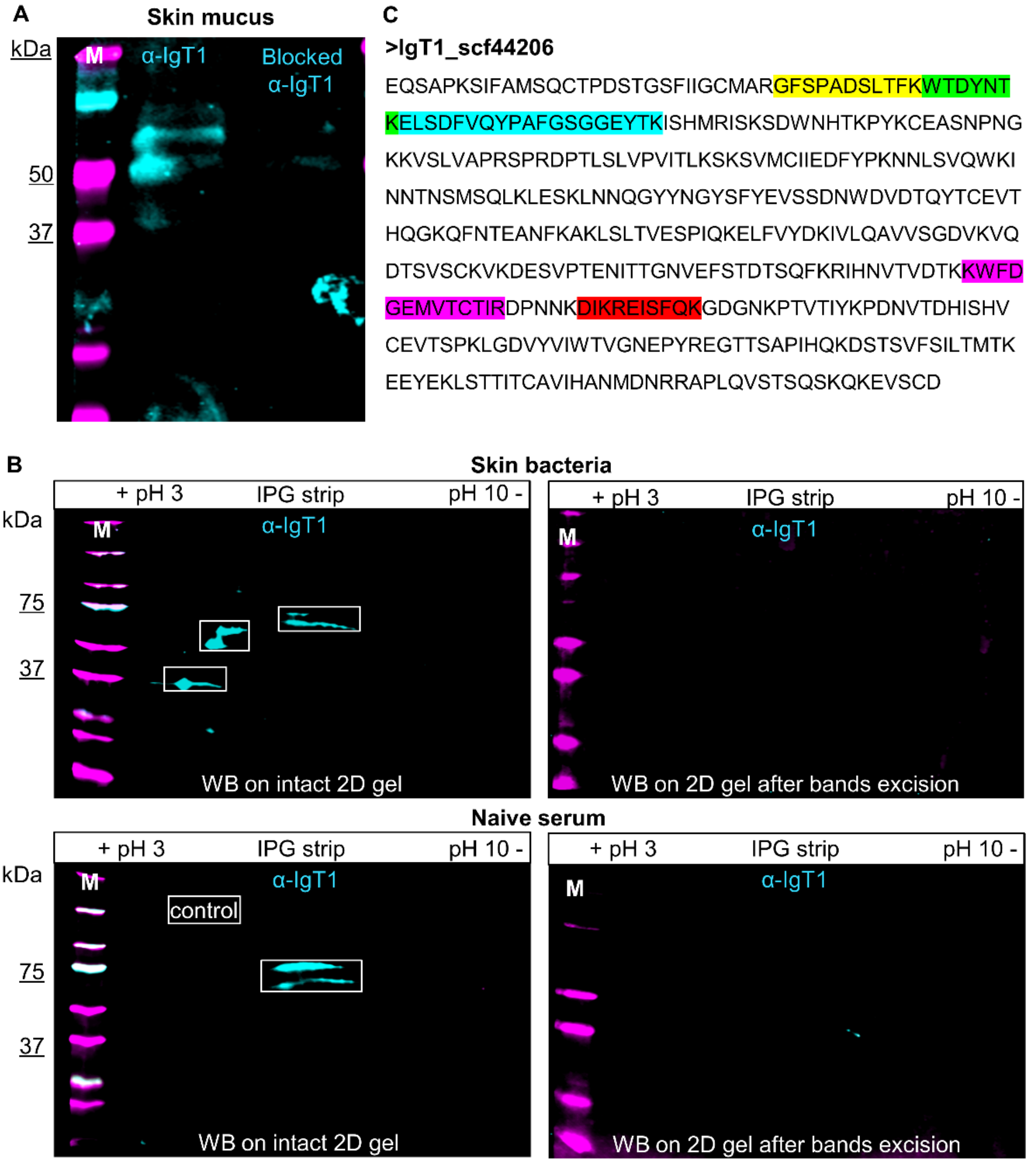

Supplementary Figure 6. Specificity of anti-IgT1 antibody. (**A**) Peptide-blocking: Total chicken IgY anti-IgT1 (α-IgT1, 10 µg/ml) were pre-incubated with 10-fold molar excess of the respective recombinant immunization peptides in tris-buffered saline (TBS) containing 5% (w/v) non-fat dry milk (NFDM, Elk) and 0.05% (v/v) tween (TBST). The reaction mixture was incubated for 1 hour at room temperature and subsequently used on western blot to detect proteins present in skin mucus (120 µg of protein/lane) using IRDye800-conjugated donkey anti-chicken (0.1 µg/ml, pseudo-coloured in cyan). Images were captured with infrared florescence imager (Odyssey). Note the absence of signal after blocking the antibodies with the respective immunization peptides. (**B**) Western blot analysis after 2D gel electrophoresis to visualize protein bands corresponding to the putative IgT1 in skin bacteria extracted from 300 µl of skin mucus (upper panels) or naïve pooled carp serum (40 µg) (lower panels). In the first-dimension electrophoresis, proteins were separated according to their isoelectric points (pI), and in the second dimension according to their molecular weight. Proteins were transferred to nitrocellulose membrane for western blot analysis as described in A. Gel slices in the area corresponding to the bands highlighted in the squares were excised from duplicate 2D gels and subjected to mass spectrometry analysis. The right panels show the results of the western blot analysis performed on the gel after excision of the putative IgT1 bands. The lack of signal confirms that the correct bands were excised prior to mass spectrometry analysis. (**C**) Peptide sequences identified by mass spectrometry in samples corresponding to proteins recognized by anti-IgT1 and excised from 2D gel as indicated in B (left panels). Proteins were prepared for MS analysis following in-gel trypsin digestion and then measured by LC-MS/MS. In total six peptides were identified for IgT1. All six peptides we identified in the 61 kDa protein band isolated from skin bacteria. From the same sample, two peptides were identified in the 50 kDa protein, and no peptide was identified in the 37 kDa protein. One peptide was identified in the 61 kDa protein band from serum. Note the first three peptides are located in the CH1τ_1 domain and due to the high similarity of this domain to CH1τ_2 and CH1µ, the peptides also give a match to IgT2 and IgM proteins.

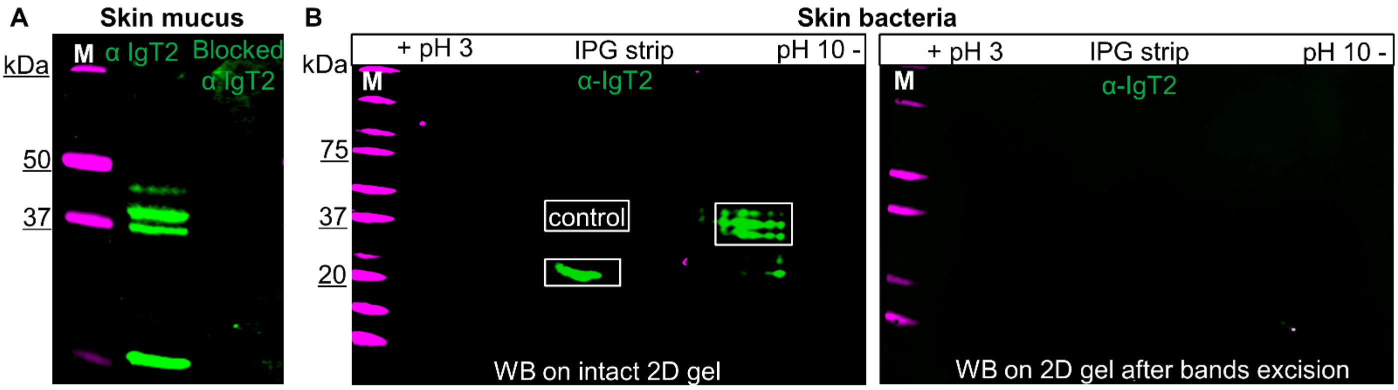

C

>IgT2_scf46259

QPSPPKSIFAMSQCTPDSTGFFTIGCMARGFSPADSLTFKWTDYAKKELSDVVQYPAFGSGEEYTKVSHVRIS
KSNWDPKNPYTCKASNSEGTSLEVKIAKAEAKAKAPDGEKPTVFIYRPDNINTDRVSHVCEVTSPKLGNVSVM
WKVGNEPYIKGTTSAPIRQNDSTSVLSILTMTKQEYEKLSTSITCAVRYGNMKDTNAPLQVSTSKSKQPELSCD

Supplementary Figure 7. (**A**) Peptide-blocking: affinity purified rabbit IgG anti-IgT2 (2.5 µg/ml) were pre-incubated with 10-fold molar excess of the recombinant immunization peptides in tris-buffered saline (TBS) containing 5% (w/v) non-fat dry milk (NFDM, Elk) and 0.05% (v/v) tween (TBST). The reaction mixture was incubated for 1 hour at room temperature and subsequently used on western blot to detect proteins present in skin mucus (120 µg of protein/lane) using IRDye800-conjugated goat anti-rabbit (0.1 µg/ml, pseudo-coloured green). Images were captured with infrared florescence imager (Odyssey). Note the absence of signal after blocking the antibodies with the immunization peptides. (**B**) Western blot analysis after 2D gel electrophoresis (as described in supplementary figure 5) to visualize IgT2 in skin bacteria extracted from 300 µl of skin mucus. Proteins were transferred to nitrocellulose membrane and western blot was performed as described in A. Note the 39 kDa protein of IgT2 around the predicted isoelectric point (pH 8.6) and the unknown 22 kDa protein around pH 6. Gel slices in the area corresponding to the bands within the squares were excised from duplicate 2D gels and subjected to mass spectrometry analysis. The right panels show the results of the western blot analysis performed on the gel after excision of the putative IgT2 bands. The lack of signal confirms that the correct bands were excised prior to mass spectrometry analysis. (**C**) Peptide sequences identified by mass spectrometry in samples corresponding to proteins recognized by anti-IgT2. In total seven peptides with a 100% match to IgT2 (scf46259) were identified by MS in samples extracted from gel slices corresponding to the 39 kDa protein, but no peptide matching IgT2 was identified in the samples derived from the 22 kDa protein.

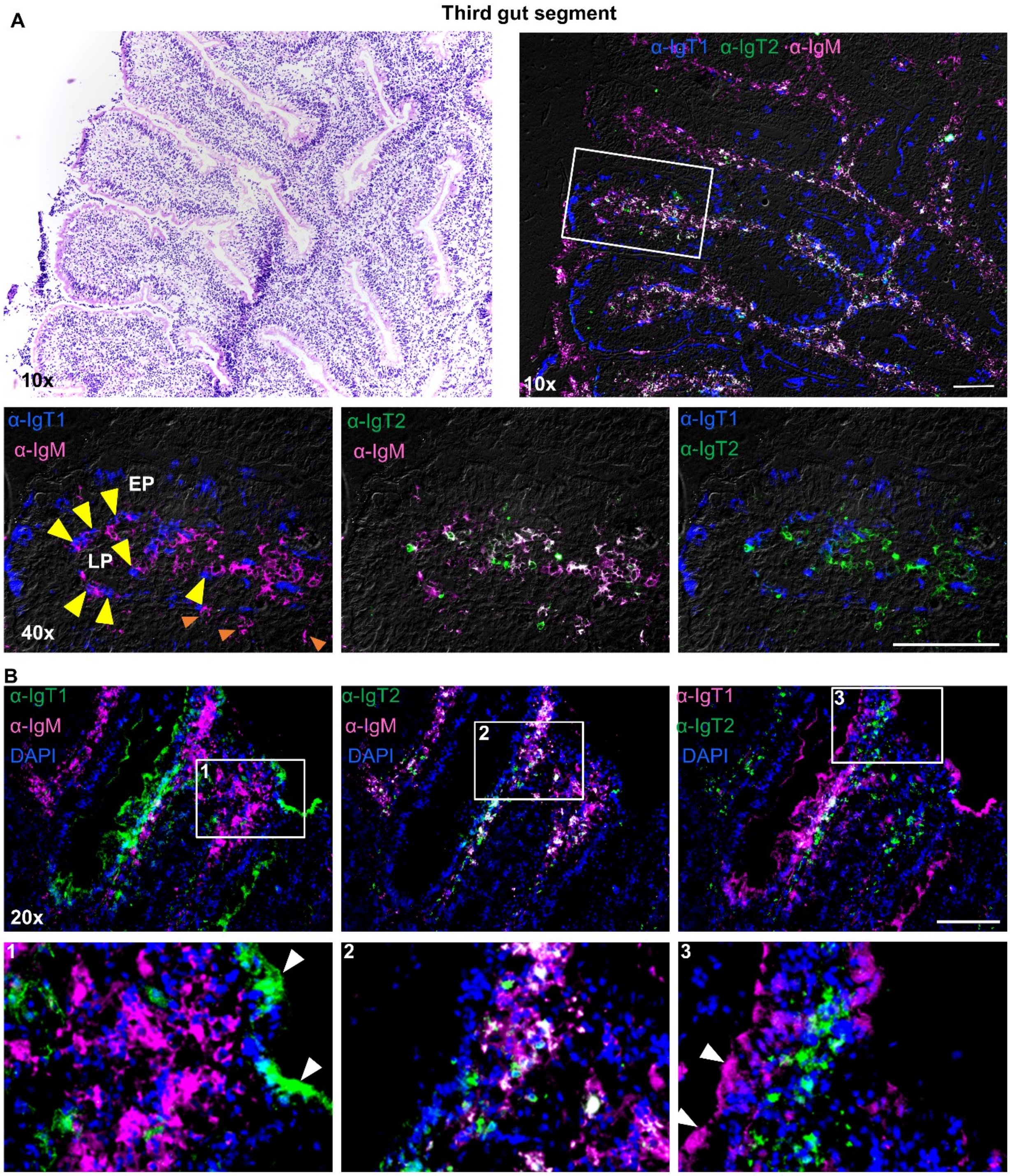

Supplementary Figure 8. Localization and tissue distribution of B cells in third gut segment. Cryosections (5 µm) of naïve third gut segment, were stained with hematoxylin and eosin and imaged at 10x magnifications (**A, upper left**). **(A, upper right)**, A consecutive section of the same gut segment was labelled to visualize IgT1$^+$ (blue), IgT2$^+$ (green) and IgM$^+$ (magenta) B cells using the same antibodies mention in figure 4. Images were acquired at 10x magnification using DM6b upright fluorescence microscope (Leica). Selected areas were imaged at 40x magnification and two-colour overlays displayed in the lower panel (**A, lower**). LP: lamina propria; EP: epithelium. **(B)** Cryosections (5 µm) of the third gut segment were labelled with the same antibodies described in figure 4 and nuclei were counterstained with DAPI. Images were acquired at 20x magnifications and distribution of two B cell types is displayed in each panel (**B, upper panel**). Insets indicated by the white squares are displayed in the lower panel showing **(B.1)** IgT1$^+$ (green) and IgM$^+$ (magenta) B cells; **(B.2)** IgT2$^+$ (green) and IgM$^+$ (magenta) B cells; **(B.3)** IgT1$^+$ (magenta) and IgT2$^+$ B cells (green). In all panels, white arrowhead points to soluble IgT1, yellow arrowhead point to IgT1$^+$ B cells in the lamina propria and orange arrowhead point to IgM$^+$ B cells in the epithelium. Secondary antibody controls are shown in **supplementary Fig 3.** Images are representatives of n = 3 fish. Images in (A and B) are from two different fish. Scale bars indicate 100 µm
